# Synthesis and Characterization of Bimetallic Copper(I) Complexes Supported by a Hexadentate Naphthyridine-Based Macrocycle Ligand

**DOI:** 10.1021/acs.inorgchem.5c00321

**Published:** 2025-04-22

**Authors:** Carlos Martínez-Ceberio, Francisco José Fernández-de-Córdova, Pablo Ríos, Orestes Rivada-Wheelaghan

**Affiliations:** Instituto de Investigaciones Químicas (IIQ), Departamento de química Inorgánica, Universidad de Sevilla, Avenida Américo Vespucio 49, Sevilla, 41092, Spain

## Abstract

Herein, we report the synthesis, characterization, and binding properties of a new ligand, *N*,*N*′-di-*tert*-butyl-3,7-diaza-1,5­(2,7)-1,8-naphthyridinacyclooctaphane (^**tBu**^ **N6**), with copper (I), Cu^I^, centers. We demonstrate the flexibility and the ability of ^**tBu**^ **N6** to adopt various conformations in solution and when coordinated to Cu^I^centers. NMR studies exhibit the labile coordination nature of Cu^I^. However, the lability of the complexes is blocked by counterion exchange, which enables the use of less coordinating solvents such as tetrahydrofuran (THF) and avoids using acetonitrile. Thus, the exchange of [BF_4_]^−^ with tetrakis 3,5-bis­(trifluoromethyl)­phenyl borate, [B­(Ar^F^)_4_]^−^, in **1·BF**_**4**_, [Cu_2_(MeCN)_2_(^tBu^N6)]­[BF_4_], generates **1·B­(Ar** ^**F**^ **)**_**4**_, which is stable in THF and reacts under a CO atmosphere to generate a *syn,syn* bis­(carbonyl) complex. This complex is sufficiently stable in solution under CO and Ar atmosphere to be characterized by NMR and IR spectroscopy, the latter revealing two stretching bands for the CO bound to the Cu^I^–centers at 2102 and 2088 cm^–1^.

## Graphical abstract

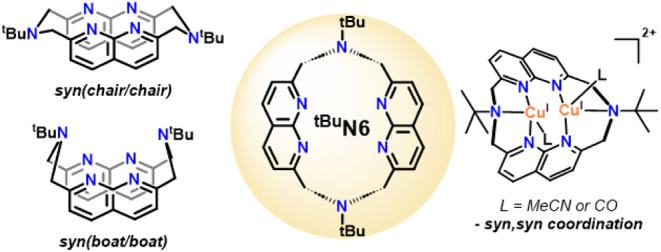

## Introduction

Since the beginning of the 21st century, there has been a renewed interest in the development of bimetallic coordination complexes, particularly those in which both metals are in close proximity.^1–5^ This resurgence is driven by the potential of such systems to exploit metal–metal cooperativity toward substrate activation pathways,^6^ chemical transformations,^7^ improved selectivity,^8,9^ and multielectron processes.^10,11^ Among the ligands explored, those based on a central 1,8–naphthyridine (Naph) core have experienced an increase in their development. This is primarily due to the *syn* disposition of two N lone pairs, which makes it an ideal choice for stabilizing binuclear systems with μ–1κN1:2κN8 coordination,^12^ while also allowing for the possibility of variation in the coordinating groups located in positions 2 and 7 of the central Naph scaffold (Figure 1). Following the pioneering biostudies carried out by Lippard et al. using Naph-based dinucleating ligands (**A**, Figure 1),^13–17^ numerous symmetrical Naph-ligands able to stabilize homobimetallic transition metal complexes have since been reported. These ligands are distinguished by the chelating groups substituted in their 2 and 7 positions.^12,18,19^ Among these, Uyeda et al. have reported dinickel-based complexes stabilized by a Naph scaffold substituted with imines (**B**, Figure 1),^9^ describing their catalytic activity in new organic transformations.^8^ Similarly, Tilley et al. have developed and studied the reactivity of several dicopper complexes stabilized by Naph ligands substituted with dipyridyl moieties (**C**, Figure 1).^4^ More recently, Broere et al. developed a Naph ligand substituted with phosphines and phosphinito groups (**D** and **E**, Figure 1)^20^ and have since studied its coordination chemistry with several transition metals and its reactivity.^21–23^ Noteworthy are the contributions of symmetrical (**F**, Figure 1) and unsymmetrical Naph-based ligands used by Bera et al.,^24–26^ including the incorporation of proton shuttle groups at the second coordination sphere to promote catalysis in monometallic species.^27,28^ All of these examples can be classified as “open-ligands” derived from a Naph core.^3^ Only recently, Colebatch et al. reported the first symmetrical Naph-based macrocyclic ligand (**G**, Figure 1),^29^ whose formation is promoted by the template effect imposed by the Ni^II^ precursor on a 2,7-bis­(di-*tert*-butylphosphinito)-Naph.^30,31^ The bimetallic complex stabilized by Naph-based macrocycle ligands has been used to study bimetallic elementary reactions.^32^ However, the phosphinito moieties limit the medium and additives compatible with this ligand for further use and studies.

**Figure 1:**
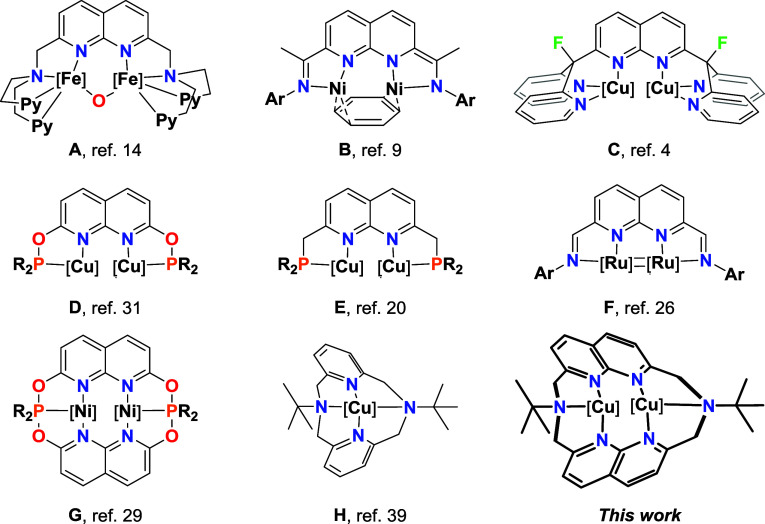
Representative examples of complexes stabilized by Naph platforms, including one featuring a pyridinaphane macrocyclic ligand and the complex described in this article.

In contrast, azamacrocycles have been demonstrated to be robust frameworks capable of stabilizing transition metal complexes and have been applied for decades in organic and inorganic catalytic transformations.^33–37^ Among the different azamacrocycles, those based on pyridinophane (**H**, Figure 1) have attracted significant attention due to their flexibility and ability to adopt various conformations when it is coordinated to a metal center due to the lability of the pendant amine groups,^38,39^ which expands the reactivity of the incorporated metal center.^40–43^ Moreover, the increased interest in these frameworks has led to improvements in their synthetic methodology.^44^ For these reasons, we have decided to attempt the synthesis of a Naph-based azamacrocycle, following procedures similar to those used for the synthesis of pyridinophane macrocycles.^44^ Thus, in this study, we report the synthesis, characterization, and copper­(I) complexation of a novel macrocyclic naphtyridinaphane ligand (^**tBu**^ **N6**), *N*,*N*′-di-*tert*-butyl-3,7-diaza-1,5 (2,7)-1,8-naphthyridinacyclooctaphane (Figure 1).

## Results and Discussion

### Ligand Synthesis and Complexation with Copper

The comprehensive synthetic pathway for the formation of ^**tBu**^ **N6** is represented in Scheme 1. The ligand precursors were synthesized according to known literature procedures.^45–47^ Once 2,7-bis­(chloromethyl)-1,8-naphthyridine (**a**) was obtained, we synthesized the 2,7-bis­(*N*,*N*′-*tert*-butylmethylene)-1,8-naphthyridine (^**tBu**^ **N-Naph**), which was characterized by High-Resolution Mass Spectrometry (HRMS) and exhibits a highly symmetric pattern in both ^1^H and ^13^C­{^1^H} Nuclear Magnetic Resonance (NMR) spectra. The reaction of compound **a** and ^**tBu**^ **N–Naph** at 80 °C in anhydrous acetonitrile solution, in the presence of a base, generates ^**tBu**^ **N6** in 35% yield after workup. The macrocyclic naphtyridinaphane ligand (^**tBu**^ **N6**), has been characterized by HRMS and by single crystal X-ray diffraction studies, where the solid-state structure exhibits a *syn*(chair/chair) conformation for the ligand (Figure S8 and Page S6). The new azamacrocyle presents two doublets (^2^ *J*_H,H_ = 14 Hz) for the methylene fragments with geminal coupling at 5.0 and 4.5 ppm. In contrast, this does not occur in macrocyclic pyridinaphane ligands (^tBu^N4), where a singlet defines the methylene fragment in their ^1^H NMR spectra.^48^ The two doublets for the methylene fragments become a broad singlet at *T* > 70 °C when the ^1^H NMR spectra of crystals of the ^**tBu**^ **N6** are recorded in a 1:1 mixture of 1,2-dichlorobenzene/CH_3_OH-*d*_*4*_ (Figure S9 and Page S6). Thus, the temperature dependence of ^**tBu**^ **N6** observed during variable temperature ^1^H NMR experiments reveals its flexibility and ability to adopt various conformations in solution.

**Scheme 1:**
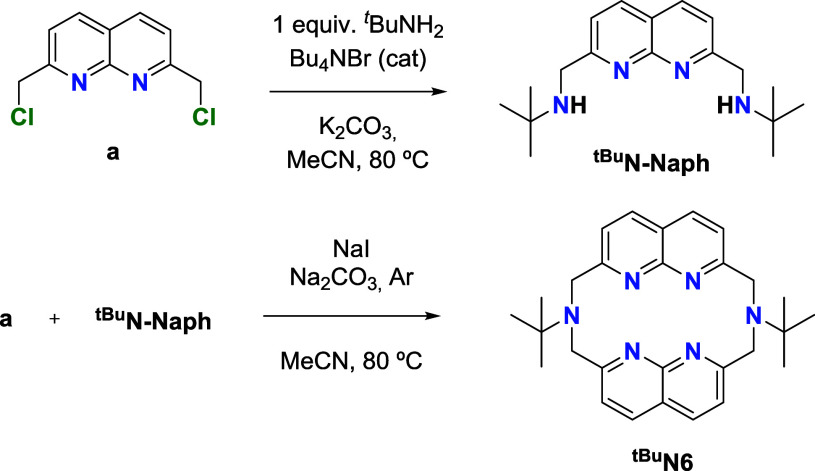
Synthesis of the Naph–Based azamacrocycle, ^**tBu**^ **N6**

Ligand ^**tBu**^ **N6** reacts with 2 equiv of tetrakis­(acetonitrile) copper­(I) tetrafluoroborate, [Cu­(MeCN)_4_]­[BF_4_], in acetonitrile solutions to generate complex **1·BF**_**4**_, [Cu_2_(^tBu^N6)­(MeCN)_2_]­[BF_4_]_2_, as the major species (87% yield, Scheme 2). Moreover, the complex can be obtained in 78% yield as orange crystals from MeCN/toluene mixtures, which are suitable for single-crystal X-ray diffraction studies (Figure 2). The ligand exhibits a *syn*(boat/boat) conformation, with both amine N atoms attached to their respective Cu^I^ atom with an elongated distance of 2.25 Å. The Cu^I^ centers are pentacoordinate, each with a MeCN molecule attached, and one of the coordinating positions involves a cuprophilic interaction with the neighboring Cu^I^ atom (Cu···Cu = 2.74 Å), as supported by DFT calculations (see Pages S27–S35).^49^ Complex **1·BF**_**4**_ is not stable in acetonitrile solutions, as evidenced by the small set of signals observed in its ^1^H NMR spectrum: 11% of another product is observed with respect to **1·BF**_**4**_, Figure S10 and Page S7). Variable-temperature ^1^H NMR analysis reveals a temperature-dependent ratio between **1·BF**_**4**_ and the compound observed in smaller ratio (Figure S39 and Pages S25–S26). Our experience on labile Cu^I^–Naph-based complexes in acetonitrile solutions^50^ leads us to consider the possibility that a Cu^I^ atom may dissociate from the structure of **1·BF**_**4**_. Thus, to a solution of ^**tBu**^ **N6** in acetonitrile, we slowly added a diluted solution containing 0.99 equiv of [Cu­(MeCN)_4_]­[BF_4_]. Under these specific reaction conditions, complex **1·BF**_**4**_ is formed at only 1%, while the major product (99%) is the one observed at 11% when complex **1·BF**_**4**_ is dissolved in CH_3_CN-*d*_*3*_. After workup, we successfully isolated and crystallized the new complex using a MeCN/toluene mixture.

**Scheme 2:**
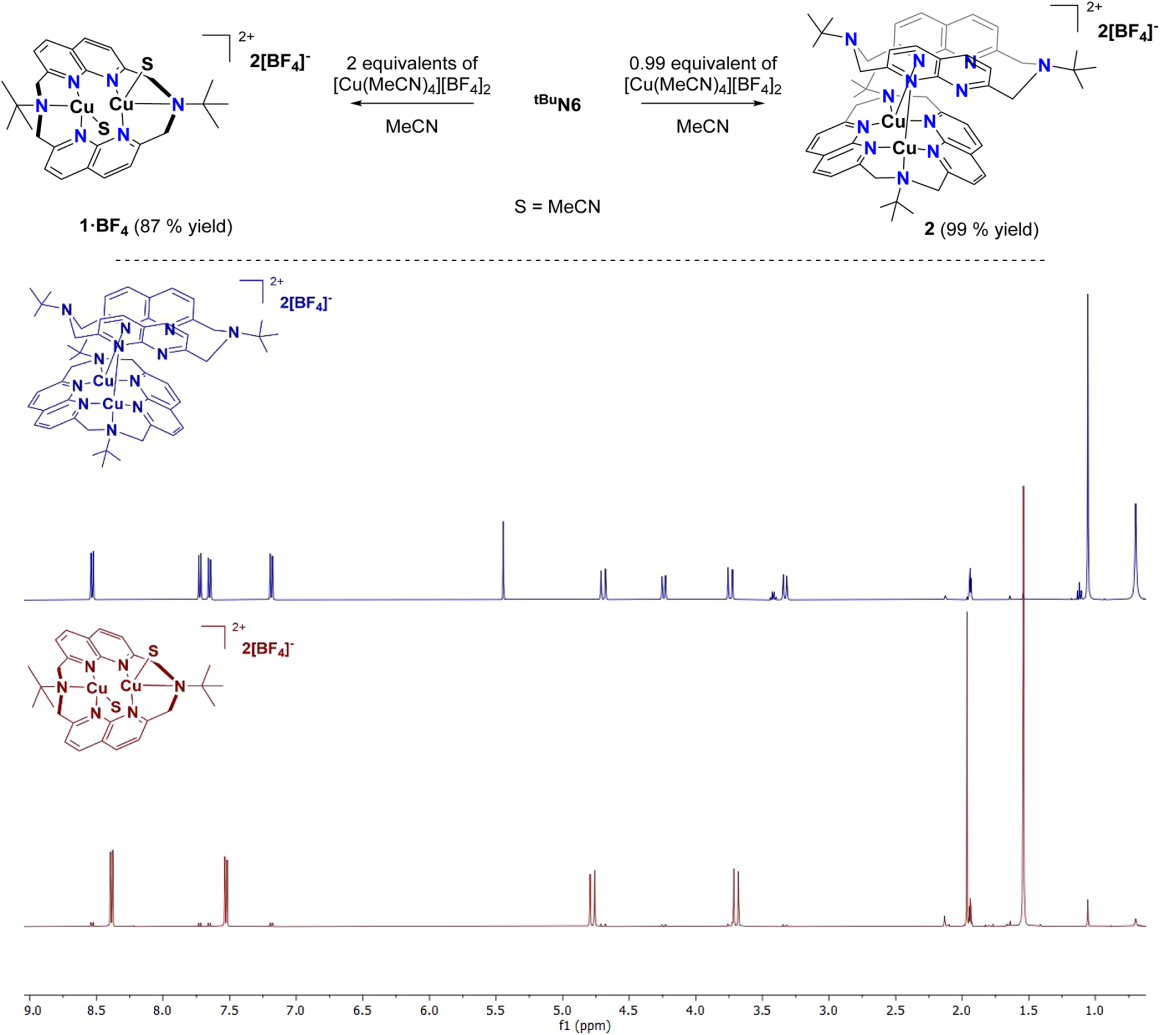
Synthesis of Complexes **1·BF**_**4**_ and **2**, and their ^1^H NMR Spectra Comparison, after Being Isolated, in CH_3_CN-*d*_3_ at 25 °C

**Figure 2:**
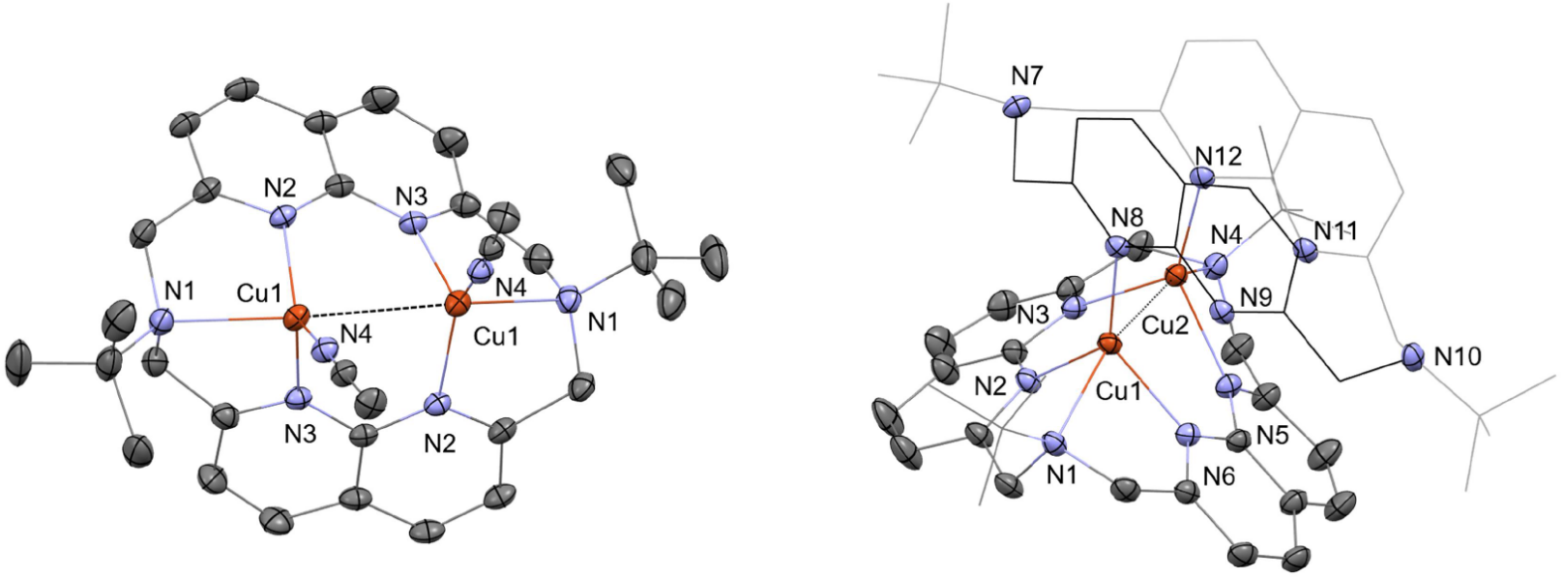
X–ray crystal structure of **1·BF**_**4**_ (left) and **2** (right). Structures are shown with 50% displacement ellipsoids. H atoms, non–coordinating ions and solvents are omitted for clarity. In complex **2**, one of the ^**tBu**^ **N6** ligands is represented as a wireframe for better visualization, with the black wireframe representing the molecule closer to the reader. The cuprophilic interaction is represented with a dashed black line. Selected bond lengths (Å) and angles (°): **1·BF**_**4**_: Cu1Cu1 = 2.7461(6); Cu1N1= 2.258(3); Cu1N2 = 2.047(1); Cu1N3 = 2.058(2); Cu1N4 = 1.898(2); N2Cu1N3 = 94.99(7); Cu1Cu1N1 = 157.59(5); N1Cu1N4 = 112.19(8); Cu1Cu1N4 = 89.93(6). **2**: Cu1Cu2 = 2.7669(7); Cu1N1 = 2.311(2); Cu1N2 2.070(2); Cu1N6 = 2.068(2); Cu1N8 = 1.975(2); Cu2N3 = 2.066(2); Cu2N4 = 2.309(3); Cu2N5 = 2.082(2); Cu2N12 = 1.983(2); N2Cu1N6 = 94.71(9); N3Cu2N5 = 94.59(9).

Interestingly, X-ray diffraction analysis shows a dicopper­(I) complex (**2**) bearing two ^**tBu**^ **N6**, [Cu_2_(^tBu^N6)_2_]­[BF_4_]_2_ ligands. In the solid-state structure, the ^**tBu**^ **N6** exhibits two different conformations: *syn*(boat/boat) for the ligand with its amine N-donors coordinated to the Cu^I^ center and *syn*(chair/chair) for the ligand with free amine N donors. The *syn*(chair/chair) conformation features shorter spatial proximity between the Naph units as compared to the *syn*(boat/boat) conformation (3.40 Å vs 5.19 Å, calculated centroid distance). The Cu^I^ atoms distance remains similar to **1·BF**_**4**_, with elongation in the Cu···N distance for the amine N–atom (2.31 Å). Analysis of the ^1^H and ^13^C­{^1^H} NMR spectra of complex **2** confirmed that the small signals observed for complex **1·BF**_**4**_ in CH_3_CN-*d*_*3*_ correspond to the presence of complex **2** in solution (Scheme 2, Figure S20–S21, Page S13).

It is important to mention that while synthesizing complexes **1·BF**_**4**_ or **2**, we noticed that when traces of chloride are present (due to salt impurities present in the ligand), a complex with the structure [Cu_2_(μ–Cl)­(^tBu^N6)]­[B­(Ar^F^)_4_]_2_ is formed in trace amount.^51^ This was confirmed by reacting complex **1·BF**_**4**_ with 1 equiv of [Bu_4_N]­[Cl] in acetonitrile solutions, which generates complex **3**, [Cu_2_(μ–Cl)­(^tBu^N6)]­[BF_4_] selectively and in high yields (Scheme 3). This complex can be purified by thorough washing with tetrahydrofuran (THF) solutions, and it crystallizes in mixtures of MeCN/toluene, yielding crystals suitable for X-ray diffraction studies (Scheme 3). In the solid-state structure, the Cu···Cu distance (2.52 Å) and the amine N–atom Cu^I^–center distances (2.18 Å) are shorter. The presence of the chloride bridging unit enhances the stability of complex **3** compared to **1** or **2** as it remains stable in the solid state when exposed to air. Moreover, **3** is the only complex identified by its HRMS peak, indicating the stabilizing effect of the μ–Cl unit in the bimetallic molecule.

**Scheme 3:**
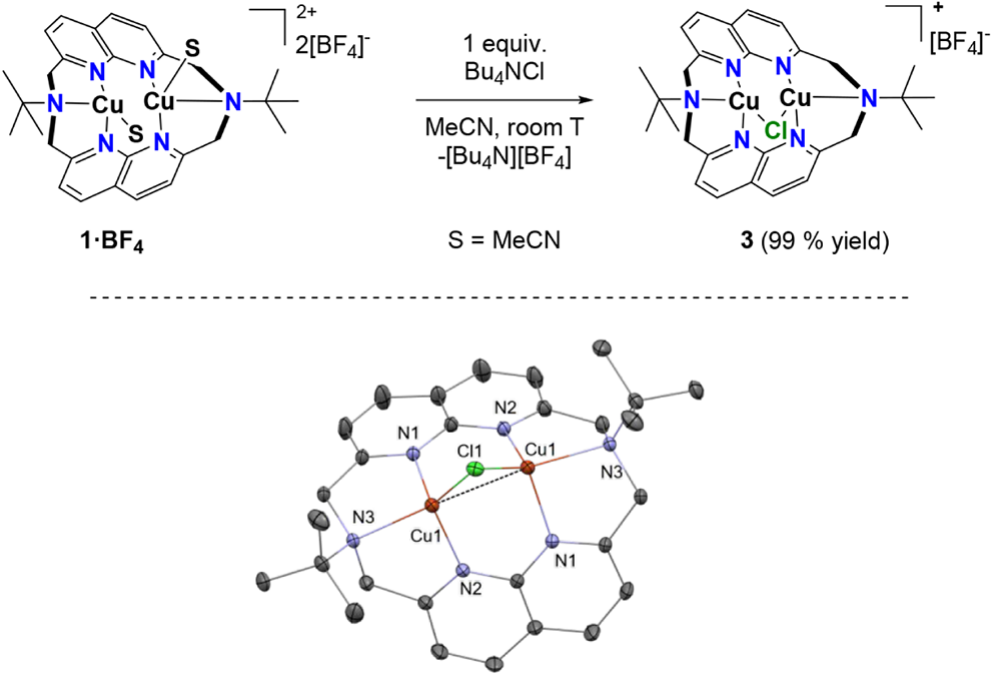
Synthesis of Complex **3** and its X-ray Crystal^a^ ^b^ ^c^ ^d^

a Structures are shown with 50% displacement ellipsoids.
b H atoms, non-coordinating ions, and solvents are omitted for clarity.
c The cuprophilic interaction is represented with a dashed black line.
d Selected bond lengths (Å) and angles (°): **3·BF**_**4**_: Cu1Cu1 = 2.5055(5); Cu1N1= 2.044(2); Cu1N2 = 2.046(2); Cu1N3 = 2.179(2); Cu1Cl1 = 2.263(3); Cu1Cl1Cu1 = 67.21; N1Cu1N2 = 99.59(9); Cu1Cu1N3 = 162.65(6); N3Cu1Cl1 = 140.86.

To collect additional data on the stability of complex **1·BF**_**4**_, we exchanged the [BF_4_] ion with [B­(Ar^F^)_4_], tetrakis-3,5–bis­(trifluoromethyl)­phenyl borate, to investigate the behavior of the complex in less coordinating solvent solutions. Therefore, complex **1·BF**_**4**_ was suspended in THF along with 1.95 equiv of [NaB­(Ar^F^)_4_]. Stirring overnight generated a suspension, which provided complex **1·B­(Ar** ^**F**^ **)**_**4**_ in 71% yield after workup. To our satisfaction, solutions of **1·B­(Ar** ^**F**^ **)**_**4**_ in MeCN–*d*_*3*_ exhibit the same set of signals as for complex **1·BF**_**4**_. Thus, the major species corresponds to **1·B­(Ar** ^**F**^ **)**_**4**_, with smaller proportions of complex **2** also present. However, in THF–*d*_*8*_ solutions, the signals corresponding to **2** are not observed. Demonstrating that acetonitrile is needed to form complex **2** from **1·[X]** due to the formation and stability of [Cu­(MeCN)_4_]­[X], X = BF_4_ or B­(Ar^F^)_4_, as a side product. Such lability has not been previously reported for other symmetrical N-substituted Naph–based ligands.^4,47^ Furthermore, while in **1·BF**_**4**_, two MeCN molecules are bound to the Cu^I^ atoms, **1·B­(Ar** ^**F**^ **)**_**4**_ exhibits a singlet for 3H in the ^1^H NMR spectrum in THF-*d*_*8*_ solutions, revealing a plausible three–center two–electron bonding of the MeCN molecule. Such bridging coordination of one MeCN molecule to both Cu^I^–centers has been previously reported for dipyridyl-Naph-stabilized dicopper­(I) complexes.^4^ Thus, **1·B­(Ar** ^**F**^ **)**_**4**_, once isolated from THF solutions, is better described as [Cu_2_(μ-MeCN)­(^tBu^N6)]­[B­(Ar^F^)_4_]_2_ (Scheme 4). Unlike other dicopper complexes stabilized by dipyridyl–Naph–ligands, **1·B­(Ar** ^**F**^ **)**_**4**_ does not transfer the aryl groups from B­(Ar^F^)_4_,^52^ remaining stable in solution after 48 h. Unfortunately, we were not able to obtain suitable crystals for X–ray diffraction studies for complex **1·B­(Ar** ^**F**^ **)**_**4**_.

**Scheme 4:**
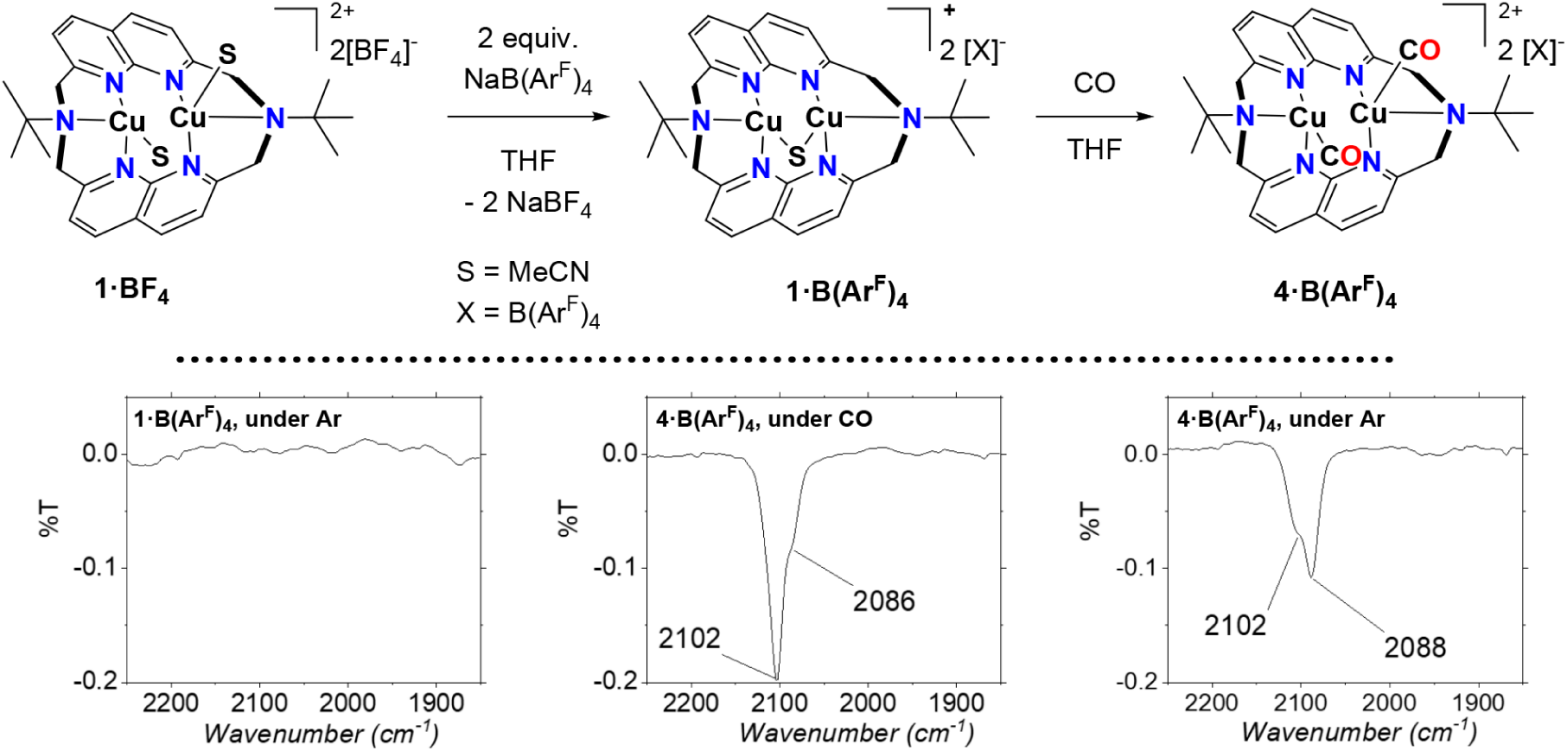
Synthesis of Complex **1·B­(Ar** ^**F**^ **)**_**4**_ and its Reactivity with CO to Generate **4·B­(Ar** ^**F**^ **)**_**4**_ (top)^a^ ^,^ [Fn sch4-fn2] ^,^ ^c^

a IR spectra of complex **1·B­(Ar** ^**F**^ **)**_**4**_ under Ar atm. (bottom left),
b
**4·B­(Ar** ^**F**^ **)**_**4**_ under CO atm. (bottom middle),
c and **4·B­(Ar** ^**F**^ **)**_**4**_ under Ar atm. (bottom right).

Since an acetonitrile molecule is displaced in complex **1·B­(Ar** ^**F**^ **)**_**4**_ when dissolved in THF, we were interested in investigating whether the remaining μ–MeCN molecule could also be displaced by a ligand–exchange reaction with a non-anionic ligand.^53^ Hence, after dissolving complex **1·B­(Ar** ^**F**^ **)**_**4**_ in THF–*d*_*8*_ and placing it in a Wilmad heavy-wall quick-pressure valve NMR tube, we performed freeze–pump–thaw cycles and backfilled the tube with 1 atm of CO. The resulting brighter solution reveals a downfield shift for the signals of the ^1^H NMR spectrum. Moreover, the ^13^C­{^1^H} NMR spectrum exhibits a new broad signal at 176 ppm (2 ppm line width), which is assigned to a CO attached to Cu^I^–center (Figure S28 and Page S18). Tilley et al. reported a chemical shift of 210 ppm for the *μ–*CO ligand of their characterized dicopper­(I) complex stabilized by 2,7-bis­(1,1-dipyridylethyl)-Naph.^53^ Fuji et al. described that the CO signal at the ^13^C NMR spectra for a series of characterized Cu^I^–CO complexes stabilized by N–tridentate ligands resonates at 175 ppm.^54^ Consequently, we describe the complex formed as a bis-carbonyl dicopper­(I) species, [Cu_2_(CO)_2_(^tBu^N6)]­[B­(Ar^F^)_4_]_2_, **4·B­(Ar** ^**F**^ **)**_**4**_. This complex was also characterized by ^1^H and ^13^C­{^1^H} NMR under Ar atm., showing the broadening of the signals in the ^1^H NMR spectrum and certain aromatic signals within the ^13^C­{^1^H} NMR spectrum. The IR spectrum of **4·B­(Ar** ^**F**^ **)**_**4**_ (19.8 mM) recorded in a THF solution confirmed the presence of two CO ligands, presenting two bands at 2102 and 2088 cm^–1^ (Scheme 4).^54^ Interestingly, the intensity of the bands varies from each other when the measurement is made under CO (2102 cm^–1^ more intense) or under Ar (2088 cm^–1^ more intense), Scheme 4, bottom. DFT calculations (see Pages S27–S31) support the assignment of two terminal CO ligands, as it predicts two CO stretches (2130 and 2120 cm^–1^ with a 6:1 intensity ratio) instead of a bridging CO moiety (one stretch, 2046 cm^–1^) or a plausible Cu­(MeCN)­Cu­(CO) complex (2113 and 2314 cm^–1^ in a 48.7:1 ratio). When replacing the CO atmosphere with Ar, decomposition occurs after 48 h, generating a gray precipitate. The instability of complex **4·B­(Ar** ^**F**^ **)**_**4**_ precludes us from obtaining its elemental analysis. Additionally, we were unable to obtain suitable crystals for X-ray diffraction analysis to confirm its structure in the solid state.^55^

## Conclusions

In summary, we report the synthesis and characterization of the new ligand *N*,*N*′-di-*tert*-butyl-3,7-diaza-1,5­(2,7)-1,8-naphthyridinacyclooctaphane, ^**tBu**^ **N6**, and its coordination chemistry with Cu^I^ centers. This manuscript demonstrates the unique properties of ligand ^**tBu**^ **N6**. The structural stability of the ligand allows it to tolerate exposure to water and air, granting it potential use for broader applications. Additionally, the flexibility of this naphthyridinaphane macrocycle is demonstrated by its ability to adopt *syn*(boat/boat) conformation across all complexes, as well as *syn*(chair/chair) conformation in complex **2**. Such fluxionality resembles those observed in pyridinaphane macrocyles (*vide supra*).

Complex **3** features a μ–Cl coordination, common in bimetallic Naph-based complexes. For **1·B­(Ar** ^**F**^ **)**_**4**_ isolated from THF solutions, we describe a three-center two-electron bonding with the MeCN molecule. However, in MeCN solutions, complexes **1·[X]** (X = BF_4_ or B­(Ar^F^)_4_) and **4·B­(Ar** ^**F**^ **)**_**4**_ present two neutral donor ligands (MeCN and CO, respectively) arranged in a *syn*– disposition. Notably, the arrangement of the carbonyl ligands in complex **4·B­(Ar** ^**F**^ **)**_**4**_, with two CO ligands in neighboring positions on the same coordination face, is particularly of interest since they resemble those proposed in C–C coupling processes on heterogeneous surfaces during CO_2_ reduction. Currently, we are expanding the family of bimetallic complexes using ^**tBu**^ **N6**.

## Experimental Section

### General Considerations

All manipulations, unless stated otherwise, were performed using Schlenk or glovebox techniques under dry argon or nitrogen atmosphere, respectively. THF, toluene, dichloromethane, and acetonitrile were freshly distilled prior to use and stored under a nitrogen atmosphere over molecular sieves (4 Å). Diethyl ether and pentane were obtained through a solvent purification system. Anhydrous deuterated solvents were purchased from Eurisotop and stored over 4 Å molecular sieves. All chemicals unless noted otherwise were purchased from major commercial suppliers (TCI, Sigma-Aldrich) and used as received.

### NMR Spectrometry

NMR spectra were recorded on Bruker Avance NEO 500, Avance NEO 400, Avance NEO 300 spectrometers. Spectral assignments were made by routine one- and two-dimensional NMR experiments where appropriate. The following abbreviations are used for describing NMR spectra: s (singlet), d (doublet), t (triplet), td (triplet of doublets), ddd (doublet of doublets of doublets), vd (virtual doublet), vt (virtual triplet), and br (broad). Chemical shifts (δ_H_, δ_C_) were quoted in parts per million (ppm) and were referenced to external SiMe_4_ (δ 0 ppm) using the residual portion solvent peaks as internal standard (^1^H NMR experiments) or the characteristic resonances of the solvent nuclei (^13^C NMR experiments).

### Electrospray Ionization High-Resolution Mass Spectrometry (ESI-HRMS)

The samples were solubilized in methanol or MeCN and then injected in direct introduction (infusion) in the mass spectrometer. Electrospray Ionization Mass Spectrometry (ESI–MS) measurements were performed on a Bruker Orbitrap Elite apparatus at the Mass Spectrometry service of the University of Seville Research, Technology and Innovation Centre.

### Elemental Analyses

These were performed by José Manuel Pérez Falcón at the Microanalytical Facility at IIQ (Instituto de Investigaciones Químicas de Sevilla), using a LECO TruSpec CHN analyzer for the determination of %C, %H, and %N.

### Vibrational Spectroscopy

FT-IR spectra were acquired using a Bruker Tensor 27 spectrometer.

### UV–visible Spectroscopy

UV–visible (UV–vis) spectroscopic instrumentation was provided by Ocean Optics. UV–vis absorption spectra were recorded using an SR-6UUV400-50 spectrometers and a DH-2000 Deuterium-Halogen light source via optical fibers (600 nm).

### Synthesis of 2,7–bis­(N,N′–tertbutylmethylene)–1,8–naphthyridine

In a flame-dried ampule, 2,7-bis­(chloromethyl)-1,8-naphthyridine (1 g, 4.4 mmol), K_2_CO_3_ (5.4 g, 40 mmol, 6 eq) and (Bu_4_N)Br (145 mg, 0.45 mmol, 0.1 eq) were suspended in 100 mL of anhydrous MeCN under Ar atmosphere. Later, *tert*–butylamine (5.14 g, 70 mmol, 16 eq) was added and the reaction mixture was heated to 80 °C and stirred vigorously overnight. After 15 h, the MeCN was removed under dynamic vacuum (from this point, the procedure was carried out under aerobic conditions). The mixture was dissolved in 100 mL of EtOAc, transferred to a separatory funnel and washed with 3 × 50 mL of HCO_3_ 10% solution. The organics were dried with Na_2_SO_4_, all solvents were removed under vacuum to give a cream-colored solid with 81% Yield (0.939 g, 3.1 mmol).^1^ H NMR (400 MHz, CHCl_3_-*d*) δ 8.07 (d, ^4^ *J*_HH_ = 8.3 Hz, 2H, CH_Naph_), 7.54 (d, ^4^ *J*_HH_ = 7.54 Hz, 2H, CH_Naph_), 4.11 (s, 4H, CH_2_N), 1.21 (s, 18H, CH_3_(*Tert–*). ^13^C­{^1^H} NMR (101 MHz, CHCl_3_–*d*) δ: 164.2 (C2, C_Naph_), 155.2 (C8, C_Naph_), 137.2 (C4, CH_Naph_), 121.6 (C3, CH_Naph_), 120.5 (C9, C_Naph_), 51.5 (C, C *(Tert–*), 49.1 (CH_2_, CN), 28.9 (CH_3_, C *(*Tert−)). ESI-HRMS (*m*/*z* pos): Found (Calc): C_18_H_29_N_4_^+^ 301.2387 (301.2392).

### Synthesis of *N*,*N*′-*tert*-butyl-2,hexaaza­[3,3]­(2,7)­pyridinophane, ^tBu^N6

A solution of 2,7-bis­(*N*,*N*′-*tert*-butylmethylene)-1,8-naphthyridine (0.35 g, 1.16 mmol) and 2,7-bis­(chloromethyl)-1,8-naphthyridine (0.151 gr, 1.16 mmol, 1 eq) in 60 mL of dry MeCN were added dropwise into a stirring suspension of Na_2_CO_3_ (1.399 g, 13.2 mmol, 20 equiv) and NaI (1.978 g, 13.2 mmol, 20 equiv) in 500 mL of dry MeCN at 80 °C, under Ar atmosphere, and was stirred during 24 h. Later, the MeCN was removed under vacuum and the solid was washed with 3 × 10 mL of cold 0 °C EtOAc. The desired product is later extracted with 5 × 10 mL of CHCl_3_ at 0 °C. The solution was dried under vacuum, and the remaining solid was washed with 3 × 2 mL of acetone at −70 °C. Finally, the solid was extracted by liquid–liquid CHCl_3_/NH_4_OH_(aq)_ extraction to give an off–white solid 35% (0.19 g, 0.41 mmol). ^1^H NMR (500 MHz, CH_3_OH–*d*_4_, CHCl_3_–*d, 1:1*) δ 8.11 (d, ^3^ *J*_HH_ 8.2 Hz, 4H, CH_Naph_), 7.69 (d, ^3^ *J*_HH_ = 8.2 Hz, 4H, CH_Naph_), 5.05 (d, ^2^ *J*_HH_ = 13.3 Hz, 4H, CH_2_N), 4.50 (d, ^2^ *J*_HH_ = 13.4 Hz, 4H, CH_2_N), 1.87 (s, 18H, CH_3_ *Tert–*). ^13^C­{^1^H} NMR (126 MHz, CH_3_OH-*d*_4_, CHCl_3_-*d, 1:1*) δ 164.07 (C2, C_Naph_), 153.16 (C8, C_Naph_), 135.73 (C4, CH_Naph_), 123.16 (C3, CH_Naph_), 118.94 (C9, C_Naph_), 58.35 (CH_2_, CN), 56.16 (C, C *Tert–*), 27.31 (CH_3_, C *Tert–*). UV–vis (MeCN), λ, nm (ε, M^–1^·cm^–1^): 227 (13500), 308 (8300). ESI-HRMS (*m*/*z* pos): Found (Calc): C_28_H_35_N_6_^+^ 455.2911 (455.2918).

### Synthesis of Complex [Cu_2_L­(MeCN)_2_]­[BF_4_]_2_, 1·BF_4_

To a suspension of ^**tBu**^ **N6** (0.090 g, 0.19 mmol) in 15 mL of MeCN, a solution of [Cu­(MeCN)_4_]­[BF_4_] (0.127 g, 0.39 mmol, 2.05 eq) in 10 mL of MeCN was added. This mixture was stirred for 30 min and then concentrated to the minimum amount of MeCN. Later, THF was added, and the precipitate that was formed was thoroughly washed with THF (5 × 10 mL), yielding a brown solid 87% yield (0.160 g, 0.16 mmol). This solid can be crystallized in a mixture of MeCN/Tol yielding up to 90%. ^1^H NMR (500 MHz, CH_3_CN–*d*_3_) δ: 8.41 (d, ^3^ *J*_HH_ = 8.3 Hz, 4H, CH_Naph_), 7.55 (d, ^3^ *J*_HH_ = 8.3 Hz, 4H, CH_Naph_), 4.80 (d, ^2^ *J*_HH_ = 17.1 Hz, 4H, CH_2_N), 3.74 (d, ^2^ *J*_HH_ = 17.1 Hz, 4H, CH_2_N), 1.99 (s, 6H, two CH_3_CN), 1.57 (s, 18H, C­(CH_3_)_3_). ^13^C­{^1^H} NMR (126 MHz, CH_3_CN-*d*_3_) δ 164.1 (C9, C_Naph_), 150.9 (CH4, C_Naph_), 139.5 (C8, C_Naph_), 123.6 (CH_3_, C_Naph_), 122.5 (C2, C_Naph_), 59.4 (C, C­(CH_3_)_3_), 57.4 (CH_2_), 25.8 (CH_3_, C­(CH_3_)_3_). UV–vis (MeCN), λ, nm (ε, M^–1^·cm^–1^): 239 (15700), 303 (8800), 365 (2900). Elemental Analysis C_32_H_40_B_2_Cu_2_F_8_N_8_ (837,418) Calculated: C 45.90, H 4.81, N 13.38; found: C 45.83; H 4.78, N 13.47.

### Synthesis of Complex [Cu_2_L­(MeCN)]­[B­(Ar^F^)_4_]_2_, 1·B­(Ar^F^)_4_

To a suspension of **1** (0.05 g, 0.06 mmol) in 15 mL of THF, a solution of [NaB­(Ar^F^)_4_] (0.10 g, 0.12 mmol, 1.95 eq) in 5 mL of THF was added. This mixture was stirred overnight, then the THF was removed under vacuum, and the solid was later extracted with 10 × 1 mL of Et_2_O. Yielding a brown solid 71% yield (0.1 g, 0.04 mmol). ^1^H NMR (500 MHz, CH_3_CN–*d*_3_) δ: 8.36 (d, ^3^ *J*_HH_ = 8.4 Hz, 4H, CH_Naph_), 7.69 (m, 8H, CH_B(ArF)4_), 7.66 (s, 4H, CH_B(ArF)4_) 7.51 (d, ^3^ *J*_HH_ = 8.4 Hz, 4H, CH_Naph_), 4.76 (d, ^2^ *J*_HH_ = 17.2 Hz, 4H, CH_2_N), 3.68 (d, ^2^ *J*_HH_ = 17.2 Hz, 4H, CH_2_N), 1.99 (s, 2 free CH_3_CN), 1.53 (s, 18H, C­(CH_3_)_3_). ^13^C­{^1^H} NMR (126 MHz, CH_3_CN–*d*_3_) δ 165.0 (C9, C_Naph_), 162.5 (Cq, C_B(ArF)4_) 151.8 (CH4, C_Naph_), 140.5 (C8, C_Naph_), 135.6 (CH, CH_B(ArF)4_), 125.7 (Cq, C_B(ArF)4_), 124.4 (CH3, C_Naph_), 123.4 (C2, C_Naph_), 118.6 (CH, CH_B(ArF)4_), 60.3 (C, C­(CH_3_)_3_), 58.3 (CH_2_), 27.2 (CH_3_, C­(CH_3_)_3_). ^1^H NMR (500 MHz, THF–*d*_8_) δ: 8.48 (d, ^3^ *J*_HH_ = 8.4 Hz, 4H, CH_Naph_), 7.78 (s, 16H, CH_B(ArF)4_), 7.63 (d, ^3^ *J*_HH_ = 8.4 Hz, 4H, CH_Naph_), 7.56 (s, 8H, CH_B(ArF)4_) 5.06 (d, ^2^ *J*_HH_ = 17.8 Hz, 4H, CH_2_N), 3.91 (d, ^2^ *J*_HH_ = 17.8 Hz, 4H, CH_2_N), 2.45 (s, CH_3_CN), 1.64 (s, 18H, C­(CH_3_)_3_). ^13^C­{^1^H} NMR (126 MHz, THF–*d*_8_) δ 164.9 (C9, C_Naph_), 163.0 (Cq, C_B(ArF)4_) 151.0 (CH4, C_Naph_), 140.8 (C8, C_Naph_), 135.8 (CH, CH_B(ArF)4_), 125.7 (Cq, C_B(ArF)4_), 124.5 (C2, C_Naph_), 123.9 (C, CH_3_ CN), 123.4 (CH3, C_Naph_), 118.4 (CH, CH_B(ArF)4_), 60.8 (C, C­(CH_3_)_3_), 59.7 (CH_2_), 27.4 (CH_3_, C­(CH_3_)_3_), 2.6 (CH_3_, CH_3_CN). UV–vis (MeCN), λ, nm (ε, M^–1^·cm^–1^): 238 (30600), 269 (21100), 306 (10400), 365 (3600). UV–vis (THF), λ, nm (ε, M^–1^·cm^–1^): 244 (260700), 267 (22700), 278 (20000), 309 (13700), 400 (2900). Elemental analysis. Calculated: C 48.06, H 2.62, N 4.17; found: C 48.18; H 2.42, N 4.23.

### Synthesis of Complex [Cu_2_ (^tBu^N6)_2_]­[BF_4_]_2_, 2

To a suspension of ^**tBu**^ **N6** (0.090 g, 0.19 mmol) in 10 mL of MeCN, a solution of [Cu­(MeCN)_4_]­[BF_4_] (0.06 g, 0.18 mmol, 0.99 equiv) in 5 mL of MeCN was added. This mixture was stirred for 30 min and then concentrated to the minimum amount of MeCN. Later, THF was added and the precipitate that formed was thoroughly washed with THF (5 × 10 mL), yielding a brown solid 76% yield (0.12 g, 0.114 mmol). This solid was put to crystallize in a mixture MeCN/Tol obtaining up to (0.095 g, 0.112 mmol) 80% crystals. ^1^H NMR (500 MHz, CH_3_CN-*d*_3_) δ: 8.56 (d, *J* = 8.4 Hz, 2H, CH_Naph_), 7.75 (d, *J* = 8.3 Hz, 2H, CH_Naph_), 7.68 (d, *J* = 8.4 Hz, 2H, CH_Naph_), 7.21 (d, *J* = 8.3 Hz, 2H, CH_Naph_), 4.72 (d, ^2^ *J*_H,H_ = 16.8 Hz, 2H, CH_2_), 4.27 (d, ^2^ *J*_H,H_ = 13.0 Hz, 2H, CH_2_), 3.77 (d, ^2^ *J*_H,H_ = 16.8 Hz, 2H, CH_2_), 3.36 (d, ^2^ *J*_H,H_ = 13.0 Hz, 2H, CH_2_), 1.08 (s, 9H, C­(CH_3_)_3_), 0.73 (s, 9H, C­(CH_3_)_3_). ^13^C­{^1^H} NMR (126 MHz, CH_3_CN-*d*_3_) δ: 163.9 (C2, C_Naph_), 163.8 (C2’, C_Naph_), 152.26 (C8, C_Naph_), 151.70 (C8’, C_Naph_), 138.91 (C4, CH_Naph_), 136.47 (C4’, CH_Naph_), 124.90 (C3, CH_Naph_), 123.69 (C9, C_Naph_), 122.73 (C3′, CH_Naph_), 120.73 (C9’, C_Naph_), 58.85 (C­(CH_3_)_3_), 58.37 (CH_2_), 57.04 (CH_2_), 56.02 (C­(CH_3_)_3_), 26.32 (C­(CH_3_)_3_), 24.89 (C­(C’H_3_)_3_). UV–vis (MeCN), λ, nm (ε, M^–1^·cm^–1^): 257 (19500), 306 (14500), 379 (4500). Elemental analysis C_56_H_68_B_2_Cu_2_F_8_N_12_ (703,97) Calculated: C 55.59, H 5.67, N 13.89; found: C 55.72, H 5.33, N 13.79.

### Synthesis of Complex [Cu_2_(Cl)^tBu^N6]­[BF_4_], 3

To a solution of **1·BF**_**4**_ (0.050 g, 0.06 mmol) in 5 mL of MeCN, tetrabutylammonium chloride (0.0164 g, 0.59 mmol, 0.99 equiv) in 2 mL of MeCN was added. This mixture was stirred for 30 min, all the MeCN was dried under vacuum, and the solid was washed with 5 × 1 mL of THF, yielding a brown solid 87% yield (0.160 g, 0.16 mmol). This solid was put to crystallize in a mixture of MeCN/Tol yielding crystals with 95% yield. ^1^H NMR (500 MHz, CH_3_CN–*d*_3_) δ: 8.18 (d, ^3^ *J*_HH_ = 8.3 Hz, 4H, CH_Naph_), 7.34 (d, ^3^ *J*_HH_ = 8.3 Hz, 4H, CH_Naph_), 4.95 (d, ^2^ *J*_HH_ = 17.3 Hz, 4H, CH_2_N), 3.83 (d, ^2^ *J*_HH_ = 17.2 Hz, 4H, CH_2_N), 1.62 (s, 18H, C­(CH_3_)_3_). ^13^C­{^1^H} NMR (126 MHz, CH_3_CN–*d*_3_) δ: 162.0 (C2, C_Naph_), 149.8 (C8, C_Naph_), 138.1 (C4, CH_Naph_), 122.7 (C9, C_Naph_), 121.9 (C3, CH_Naph_), 59.5 (CH_2_N), 26.9 (C­(CH_3_)_3_). The signal for C­(CH_3_)_3_, could not be found in the spectrum. UV–vis (MeCN), λ, nm (ε, M^–1^·cm^–1^): 233 (8300), 271 (6100), 311 (2900), 377 (400). ESI–HRMS (*m*/*z* pos): Found (Calc): C_28_H_34_N_6_ClCu_2_^+^ 615.1137 (615.1120). Elemental Analysis C_28_H_34_BCu_2_F_4_N_6_Cl (703,96) Calculated: C 47.77, H 4.87, N 11.94; found: C 47.72; H 4.714, N 12.13.

### Synthesis of Complex [Cu_2_(^tBu^N6)­(CO)]­[B­(Ar^F^)_4_]_2_, 4·B­(Ar^F^)_4_

Complex **1·B­(Ar** ^**F**^ **)**_**4**_ (0.04 g, 0.017 mmol) was dissolved in 0.4 mL of THF–d_8_ and transferred to a Heavy Wall Quick Pressure Valve NMR Tube (Wilmad) under Ar. Later, the solution was subjected to freeze–pump–thaw and filled with 1 atm. of CO gas, a moment in which the solution turns from brown to orange (yellow when diluted). The complex formed, exhibits the NMR signals shown in Figures S27 and S28. Complex **4·B­(Ar** ^**F**^ **)**_**4**_, under CO atmosphere: ^1^H NMR (500 MHz, THF–*d*_8_) δ: 8.69 (d, ^3^ *J*_HH_ = 8.3 Hz, 4H, CH_Naph_), 7.83 (d, ^3^ *J*_HH_ = 8.4 Hz, 4H, CH_Naph_), 7.78 (s, 16H, CH_B(ArF)4_), 7.56 (s, 8H, CH_B(ArF)4_) 5.14 (d, ^2^ *J*_HH_ = 17.5 Hz, 4H, CH_2_), 3.97 (d, ^2^ *J*_HH_ = 17.5 Hz, 4H, CH_2_), 2.00 (s, CH_3_CN), 1.63 (s, 18H, C­(CH_3_)_3_). ^13^C­{^1^H} NMR (126 MHz, THF-*d*_8_) δ: 176.1 (br, CO), 166.9 (C9, C_Naph_), 163.0 (Cq, C_B(ArF)4_) 152.2 (C8, C_Naph_), 142.9 (CH4, C_Naph_), 135.8 (CH, CH_B(ArF)4_), 130.2 (Cq, C_B(ArF)4_)), 125.8 (Cq, C_B(ArF)4_), 125.2 (C2, C_Naph_), 123.9 (CH3, C_Naph_), 118.4 (CH, CH_B(ArF)4_), 118.1 (Cq, free CH_3_ CN), 62.3­(C, C­(CH_3_)_3_), 59.3­(CH_2_), 27.2 (br, CH_3_, C­(CH_3_)_3_), 1.1 (CH_3_, CH_3_CN). UV–vis (THF), λ, nm (ε, M^–1^·cm^–1^): 272 (23700), 303 (17000), 402 (3900). FT–IR (THF solution), cm^–1^: 2102 (sharp, intense, CO), 2088 (sharp, medium intensity, CO). Complex **4·B­(Ar** ^**F**^ **)**_**4**_, under Ar atmosphere: ^1^H NMR (500 MHz, THF–*d*_8_) δ: 8.69 (br, 4H, CH_Naph_), 7.83 (s, 4H, CH_Naph_ + 16H, CH_B(ArF)4_), 7.61 (s, 8H, CH_B(ArF)4_), 5.14 (d, ^2^ *J*_HH_ = 17.9 Hz, 4H, CH_2_), 3.98 (d, ^2^ *J*_HH_ = 17.9 Hz, 4H, CH_2_), 2.13 (s, CH_3_CN), 1.66 (s, 18H, C­(CH_3_)_3_). ^13^C­{^1^H} NMR (126 MHz, THF–*d*_8_) δ: 176.1 (br, CO), 166.5 (br, C9, C_Naph_), 163.0 (Cq, C_B(ArF)4_) 152.1 (C8, C_Naph_), 142.2 (br, CH4, C_Naph_), 135.8 (CH, CH_B(ArF)4_), 130.2 (Cq, C_B(ArF)4_)), 125.8 (Cq, C_B(ArF)4_), 125.0 (C2, C_Naph_), 123.8 (CH_3_, C_Naph_), 118.4 (CH, CH_B(ArF)4_), 119.1 (Cq, free CH_3_ CN), 61.6 (C, C­(CH_3_)_3_), 59.1 (CH_2_), 27.2 (br, CH_3_, C­(CH_3_)_3_), 0.68 (CH_3_, CH_3_CN). UV–vis (THF), λ, nm (ε, M^–1^·cm^–1^): 280 (22300), 303 (16800), 396 (3700). FT–IR (THF solution), cm^–1^: 2102 (sharp, medium intensity, CO), 2088 (sharp, intense, CO).

## Supplementary Material



## Notes

Experimental spectra, and crystallographic data (PDF)
