# Supplementary material for: Synthesis and Characterization of Bimetallic Copper(I) Complexes Supported by a Hexadentate Naphthyridine-Based Macrocycle Ligand
Source: Inorg Chem. 2025 Apr 22;64(17):8630–8. doi: 10.1021/acs.inorgchem.5c00321 (PMC12135031; doi:10.1021/acs.inorgchem.5c00321)
Supplement: Supplementary file 1 [file ic5c00321_si_001.pdf]

Supporting Information

Synthesis and Characterization of Bimetallic Copper (I) Complexes Supported by a Hexadentate Naphthyridine-Based Macrocycle Ligand

Carlos Martínez–Ceberio[a], Francisco José Fernández de Córdova[a], Pablo Ríos[a] and Orestes Rivada–Wheelaghan[a]*

[a] *Departamento de Química Inorgánica – Instituto de Investigaciones Químicas, Universidad de Sevilla, C/Américo Vespucio 49, 41092 Sevilla, Spain*

E–mail: orivada@us.es

Table of contents

1. Experimental spectra of 2,7–bis(N,N′–tertbutylmethylene)–1,8–naphthyridine…..………………………………………………………………S2
2. Experimental spectra of N,N′–tertbutyl-2,hexaaza[3,3](2,7)pyridinophane, **L**………………………………….……………………………………………S4
3. Experimental spectra of complex **1·BF$_4$**, $[Cu_2(MeCN)_2L][BF_4]_2$,……….………S7
4. Experimental spectra of complex **1·B(Ar^F)$_4$**, $[Cu_2(\mu\text{–}MeCN)L][B(Ar^F)_4]_2$,…..S9
5. Experimental spectra of complex **2**, $[Cu_2L_2][BF_4]_2$…………………….……….……S13
6. Experimental spectra of complex **3**, $[Cu_2(\mu\text{–}Cl)L][BF_4]$…………….…….….…S15
7. Experimental spectra of complex **4·B(Ar^F)$_4$**, $[Cu_2(CO)_2L][B(Ar^F)_4]_2$……..…S17
8. Distribution between **1·BF$_4$** and **2** in acetonitrile solutions……….………..…S25
9. Computational Details………………………………….………….……..…S27
10. Crystallographic Details……………………………….…………….……..…S36

2,7–bis(N,N′–tertbutylmethylene)–1,8–naphthyridine

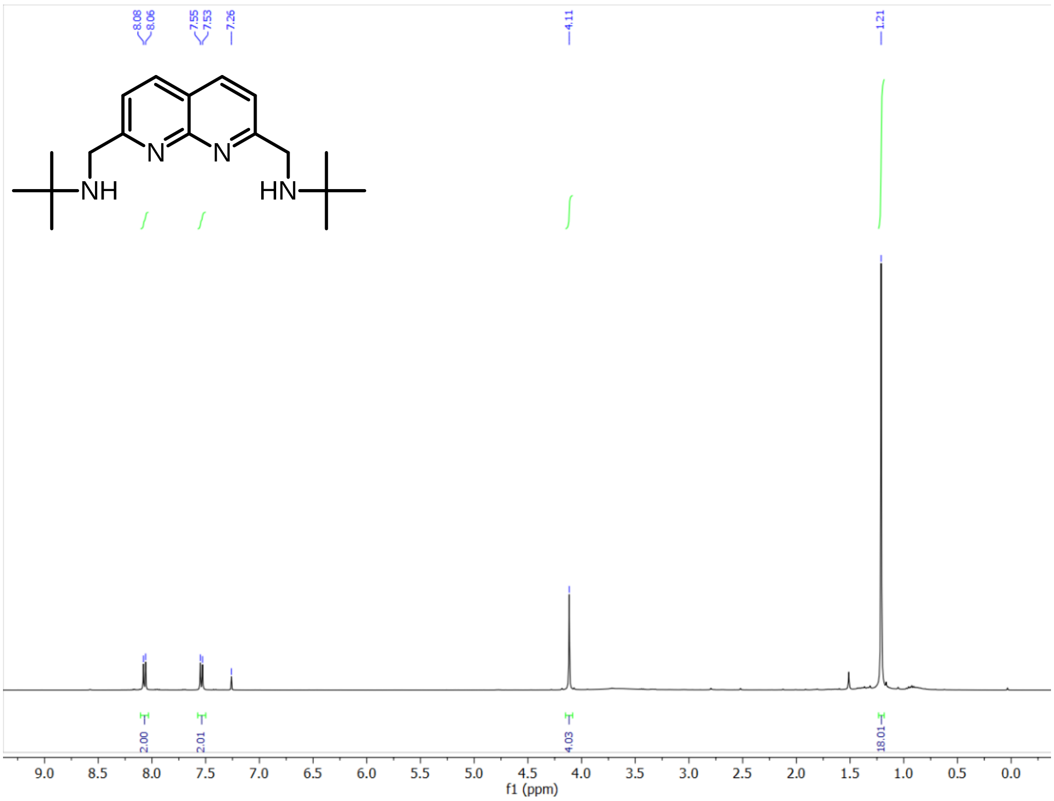

Figure S1. ^{1}H NMR spectrum of 2,7–bis(N,N′–tertbutylmethylene)–1,8–naphthyridine in $CHCl_3$–*d* at 25 ºC.

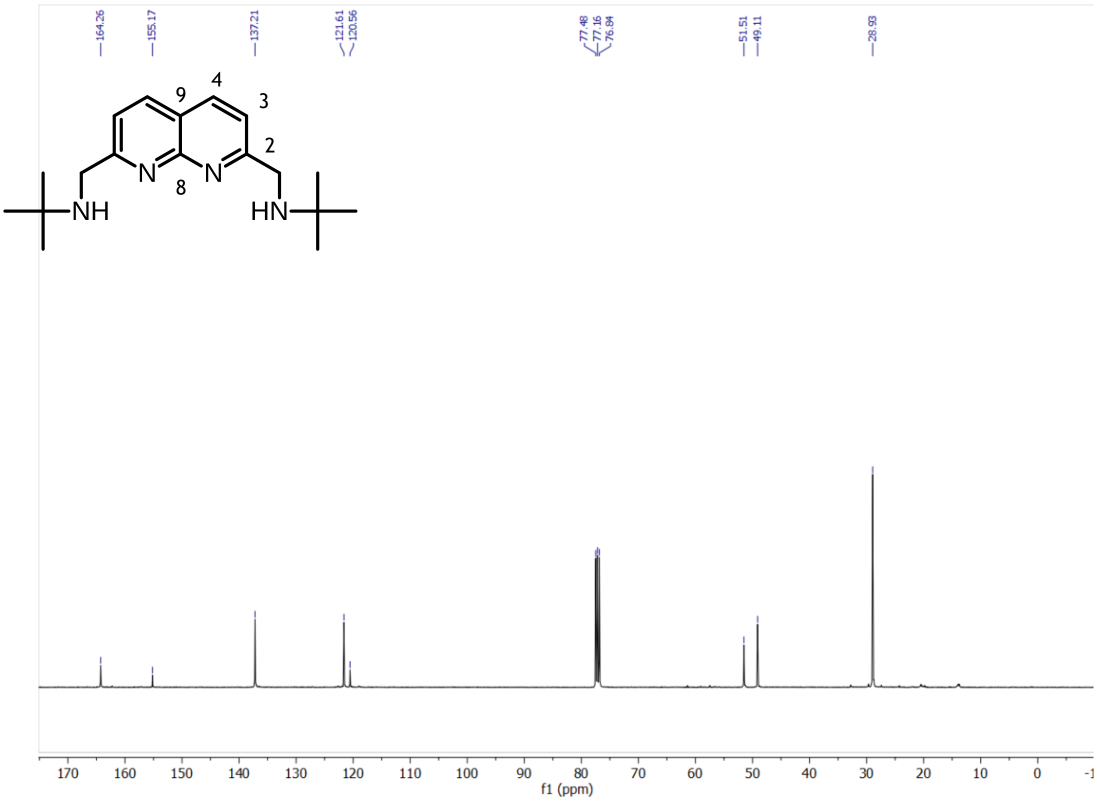

Figure S2. $^{13}C\{^{1}H\}$ NMR spectrum of 2,7–bis(N,N′–tertbutylmethylene)–1,8–naphthyridine in $CHCl_3$–*d* at 25 ºC..

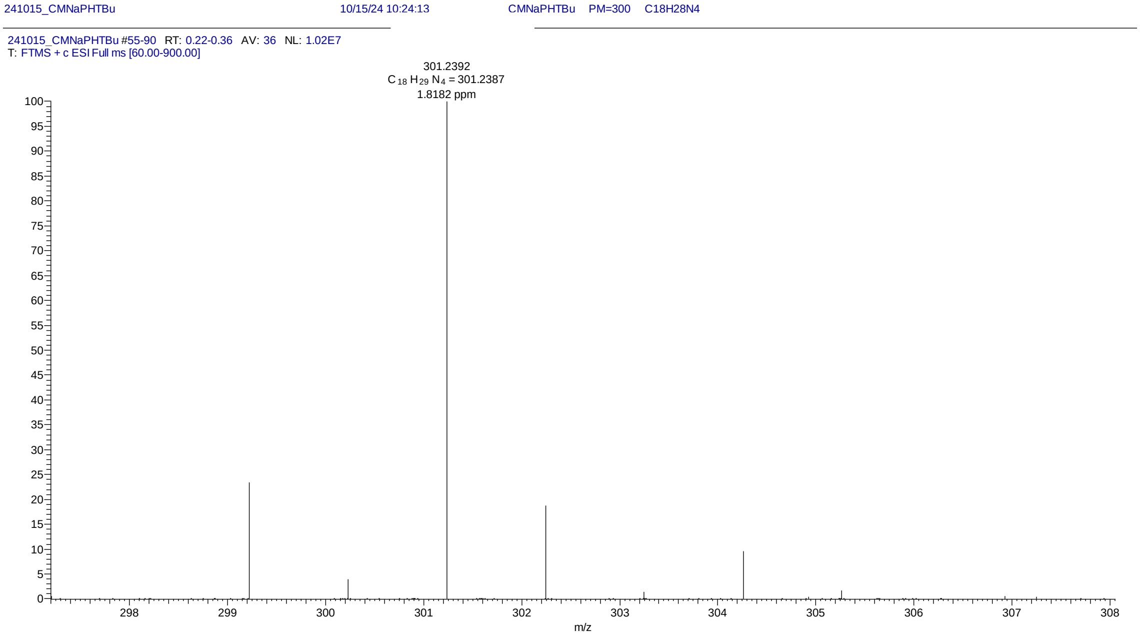

Figure S3. Experimental ESI–MS spectrum of 2,7–bis(N,N′–tertbutylmethylene)–1,8–naphthyridine.

N,N′–tertbutyl–2,hexaaza[3,3](2,7)pyridinophane, ^{tBu}N6.

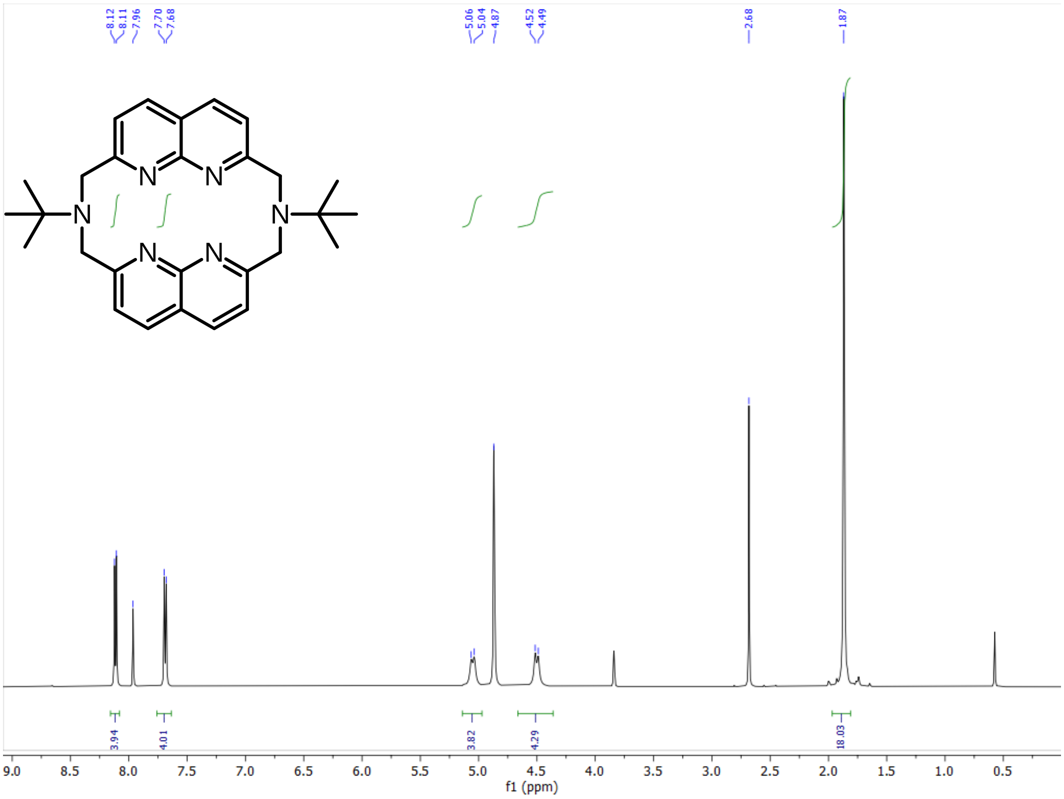

Figure S4. ^{1}H NMR spectrum of **^{tBu}N6** in CH_3OH–*d*$_4$, $CHCl_3$–*d*, *1:1*, at 25 ºC.

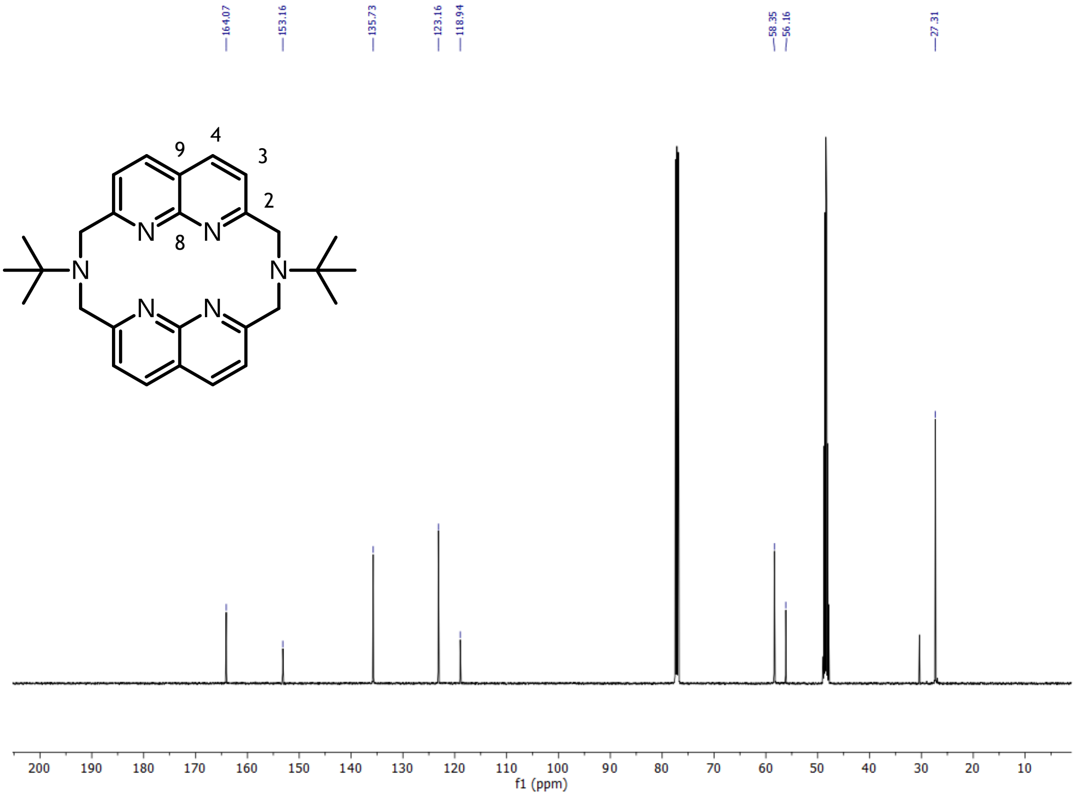

Figure S5. ^{13}C{^{1}H} NMR spectrum of **^{tBu}N6** in CH_3OH–*d*$_4$, $CHCl_3$–*d*, *1:1*, at 25 ºC.

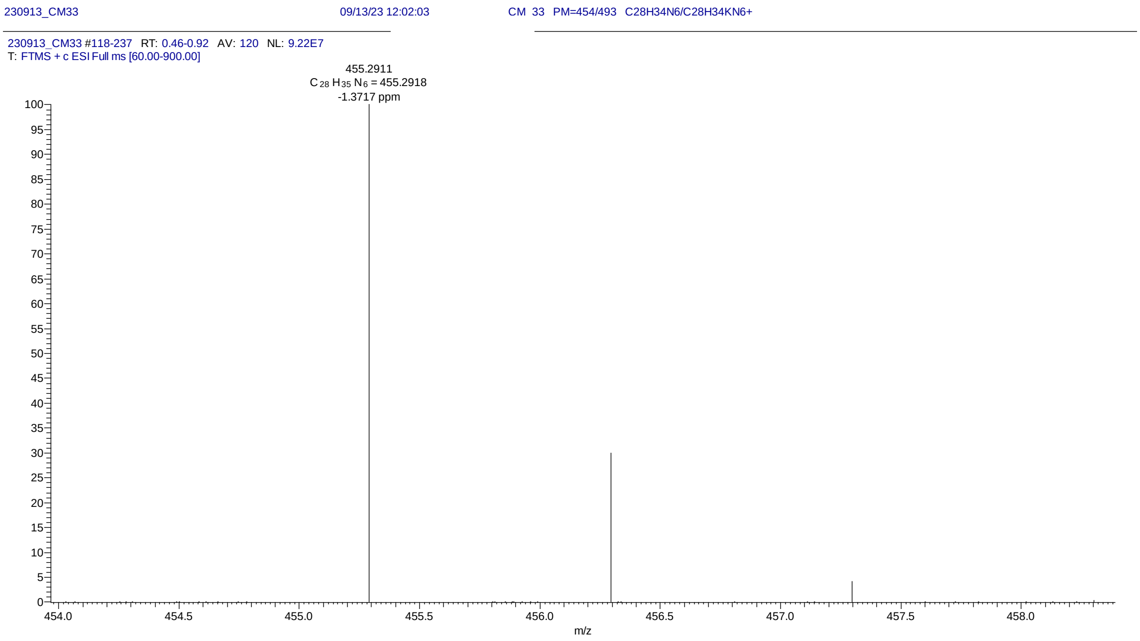

Figure S6. Experimental ESI–MS spectrum of **^{tBu}N6**.

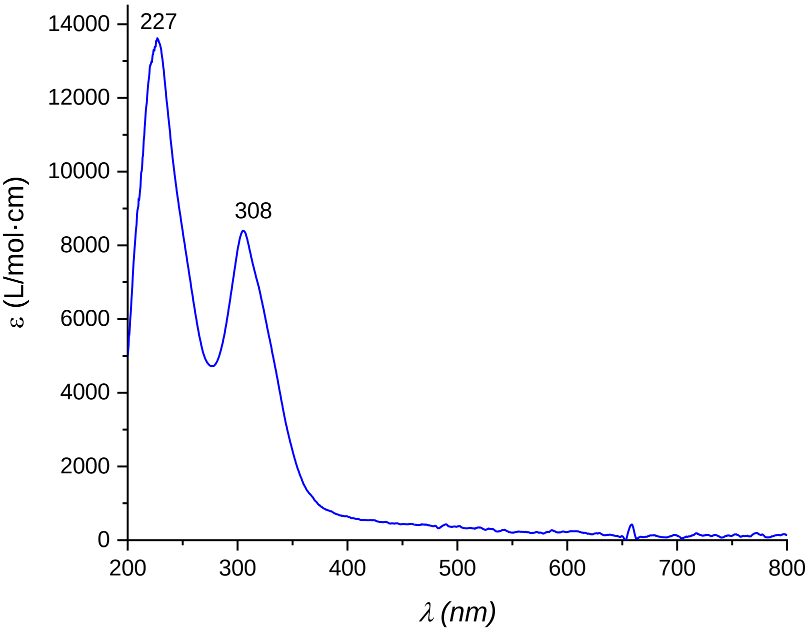

Figure S7. UV–visible spectrum of **^{tBu}N6** in CH_3CN, the thickness of the quartz cell is 1 cm (90 μM).

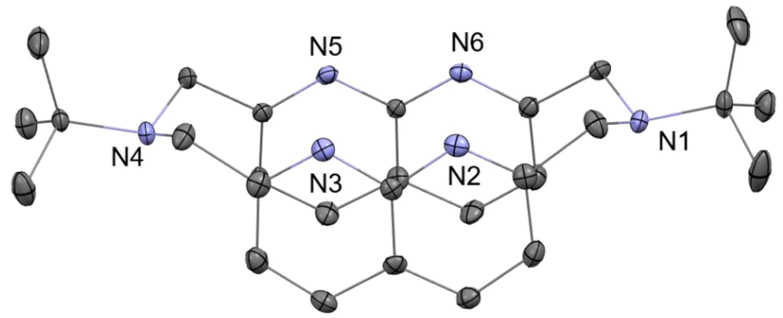

Figure S8. X–ray crystal structure of **^{tBu}N6** (left) shown with 50 % displacement ellipsoids. H atoms are omitted for clarity.

Variable Temperature ^{1}H NMR Experiments. 7 mg of crystals of **^{tBu}N6** where dissolved in 0.4 mL of a mixture $C_5H_4Cl_2$/CH_3OH-d_4, 1:1, (1,2-dichlorobenzene/deuterated methanol) and the solution was transferred to a screw cap NMR tube. The different ^{1}H NMR spectra where recorded at a specific temperature with increments of 10° C, from 30° to 80° C, and waiting 15 min after each temperature was stabilized.

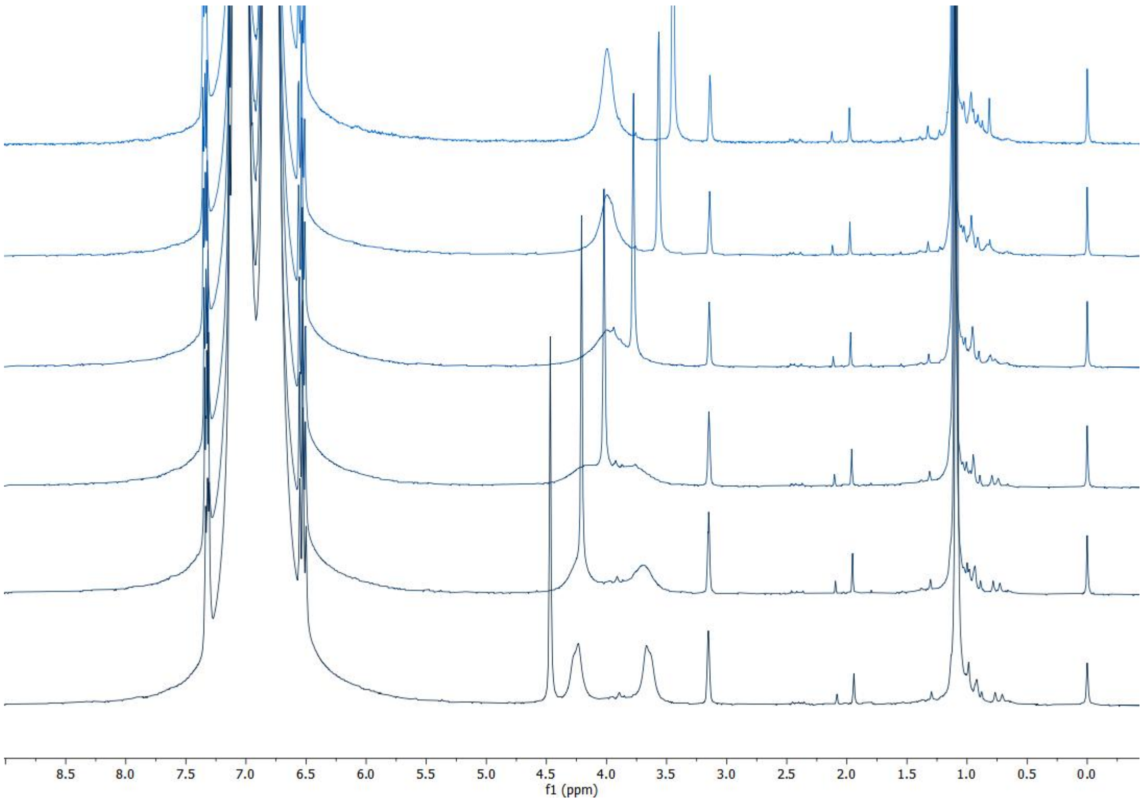

Figure S9. ^{1}H NMRs spectra of **^{tBu}N6** in $C_5H_4Cl_2$/CH_3OH-d_4 (1:1) at different temperatures ranging from 30° (bottom) to 80° C (top) with a 10° C difference, under Ar atm. The broad aromatic signals belong to $C_5H_4Cl_2$ and the singlet shifting from 4.5 ppm to 3.5 ppm while the temperature increase belongs to CH_3OH-d_4.

Complex [Cu2(tBuN6)(MeCN)2][BF4]2, 1·BF4:

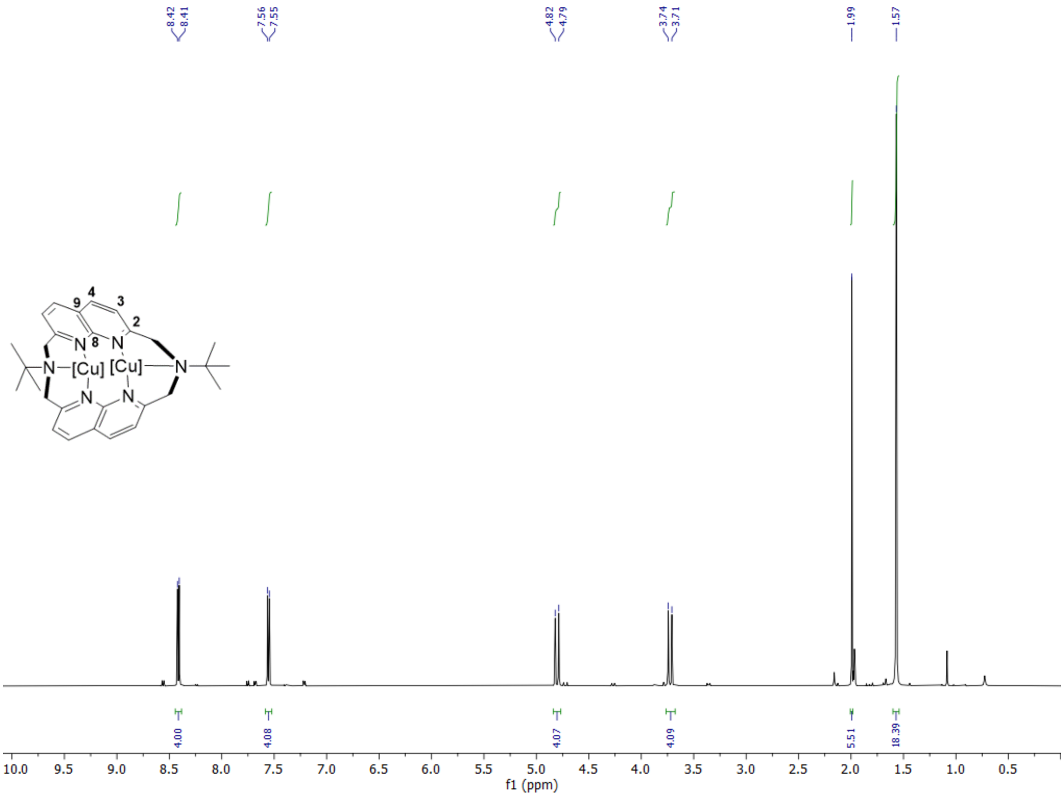

Figure S10. ^{1}H NMR spectrum of **1·BF4** in CH_3CN–d_3 at 25 ºC.

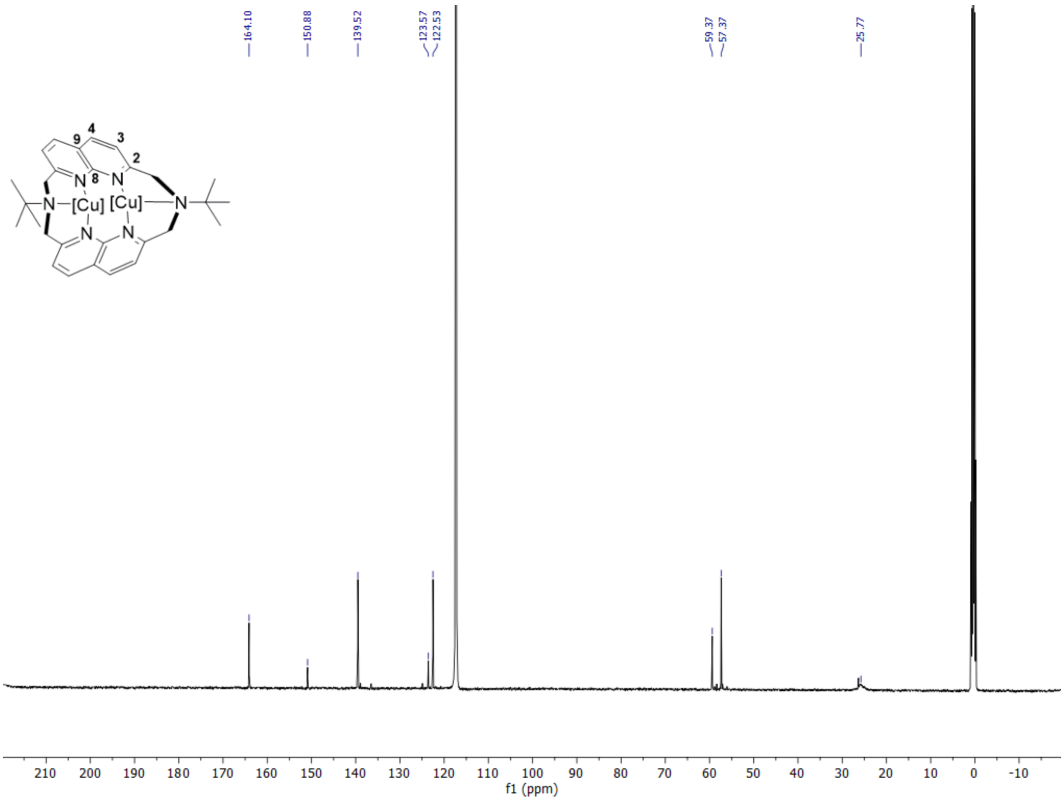

Figure S11. $^{13}C\{^{1}H\}$ NMR spectrum of **1·BF4** in CH_3CN–d_3 at 25 ºC.

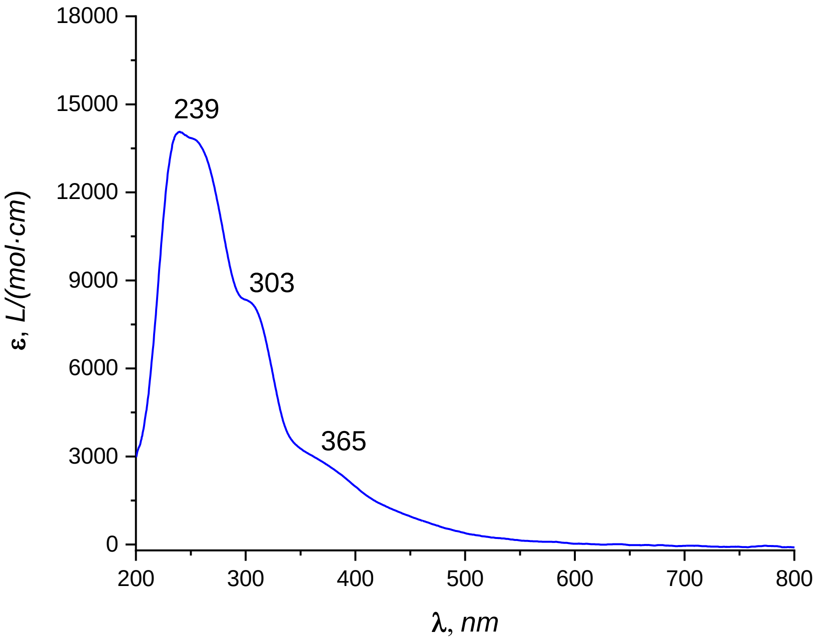

Figure S12. UV–visible spectrum of **1·BF₄** in CH_3CN, the thickness of the quartz cell is 1 cm (90 μM).

Complex $[Cu_2(^{tBu}N6)(MeCN)][B(Ar^F)_4]_2$, 1·$B(Ar^F)_4$:

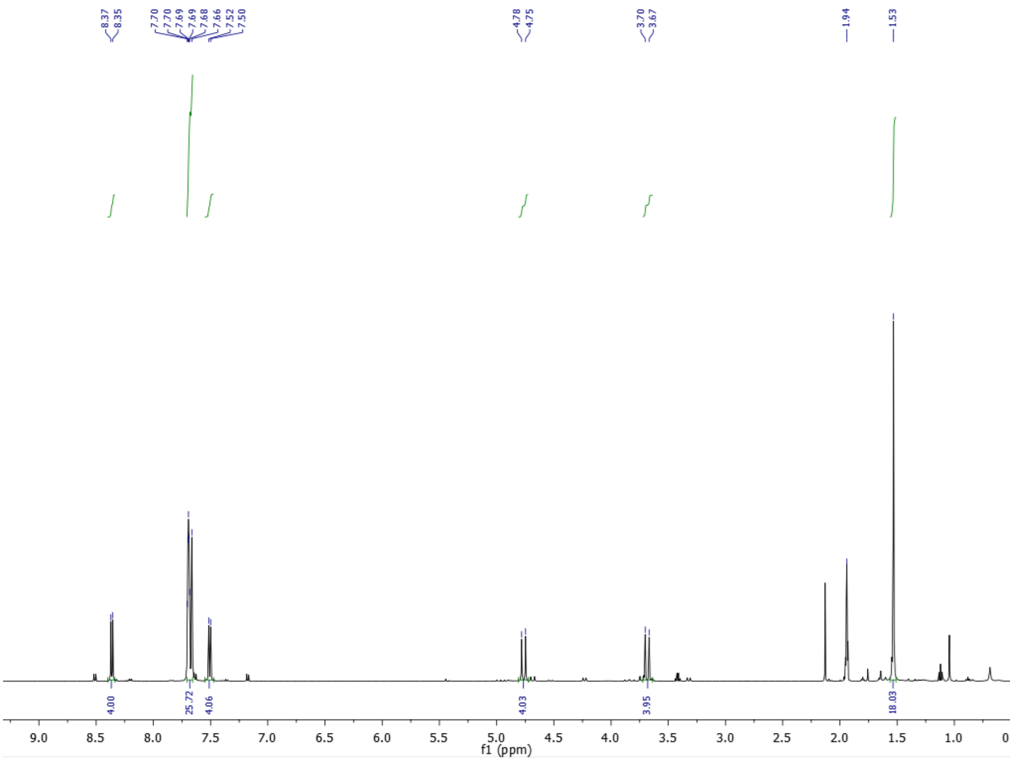

Figure S13. ^{1}H NMR spectrum of **1·$B(Ar^F)_4$** in CH_3CN–d_3 at 25 ^oC. The smaller peaks between 3.5 and 9 ppm match those of complex **2** (Figure S16).

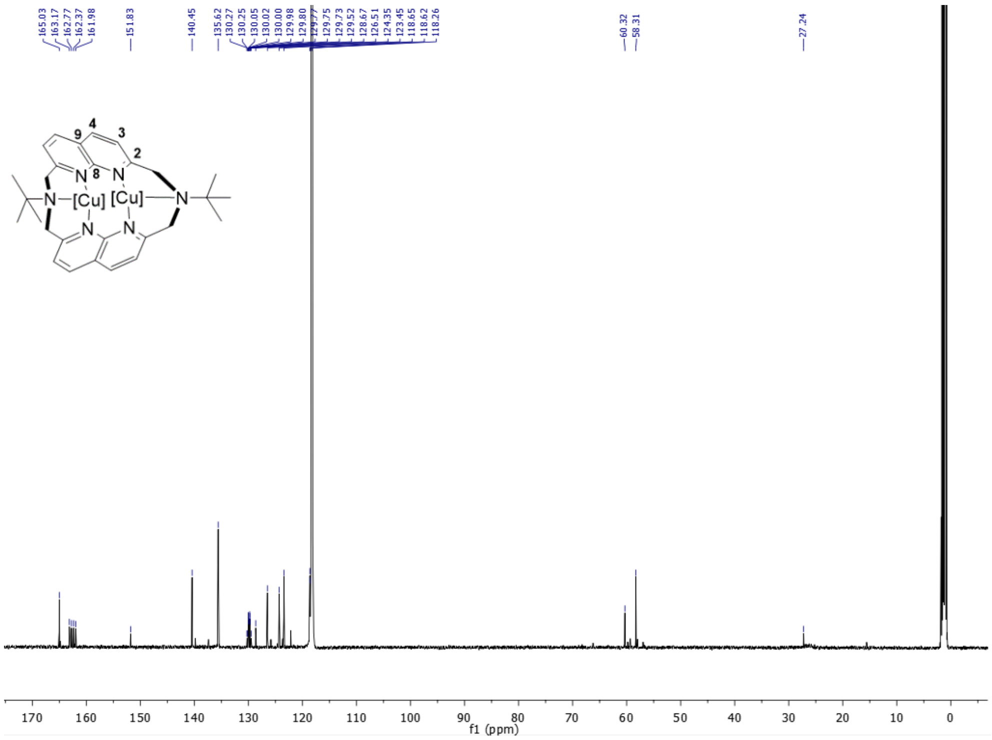

Figure S14. $^{13}C\{^{1}H\}$ NMR spectrum of **1·B(Ar^{F})$_4$** in CH_3CN–d_3 at 25 ºC. The smaller peaks match those of complex **2** (Figure S17).

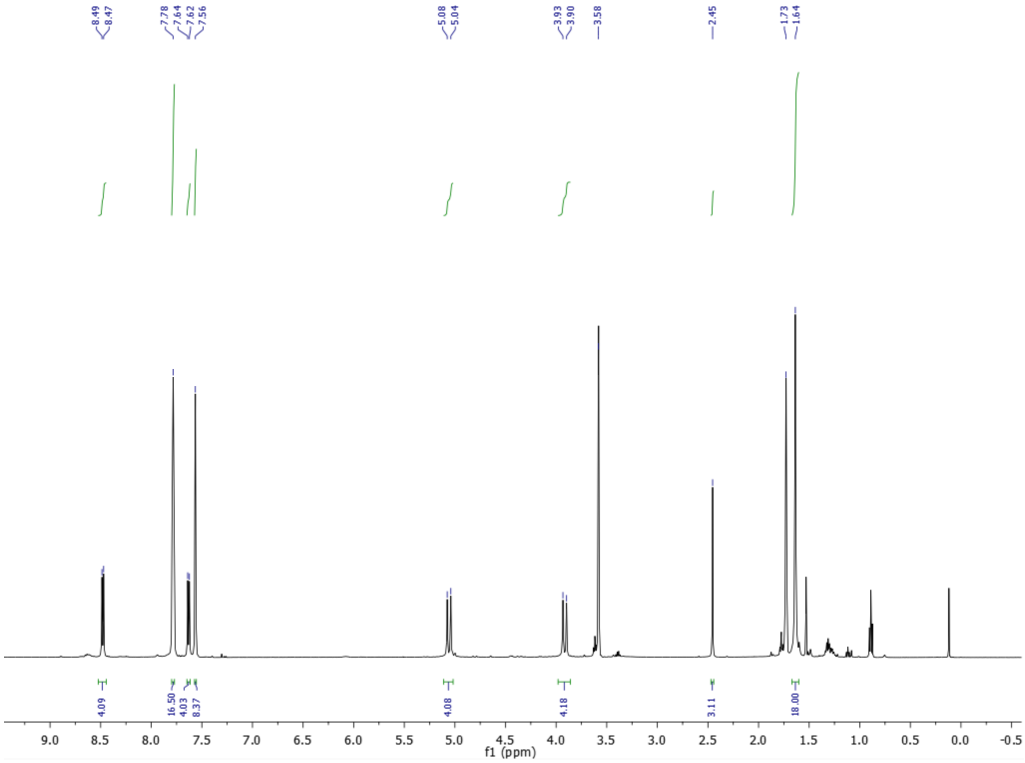

Figure S15. ^{1}H NMR spectrum of **1·B(Ar^{F})$_4$** in THF–d_8 at 25 ºC.

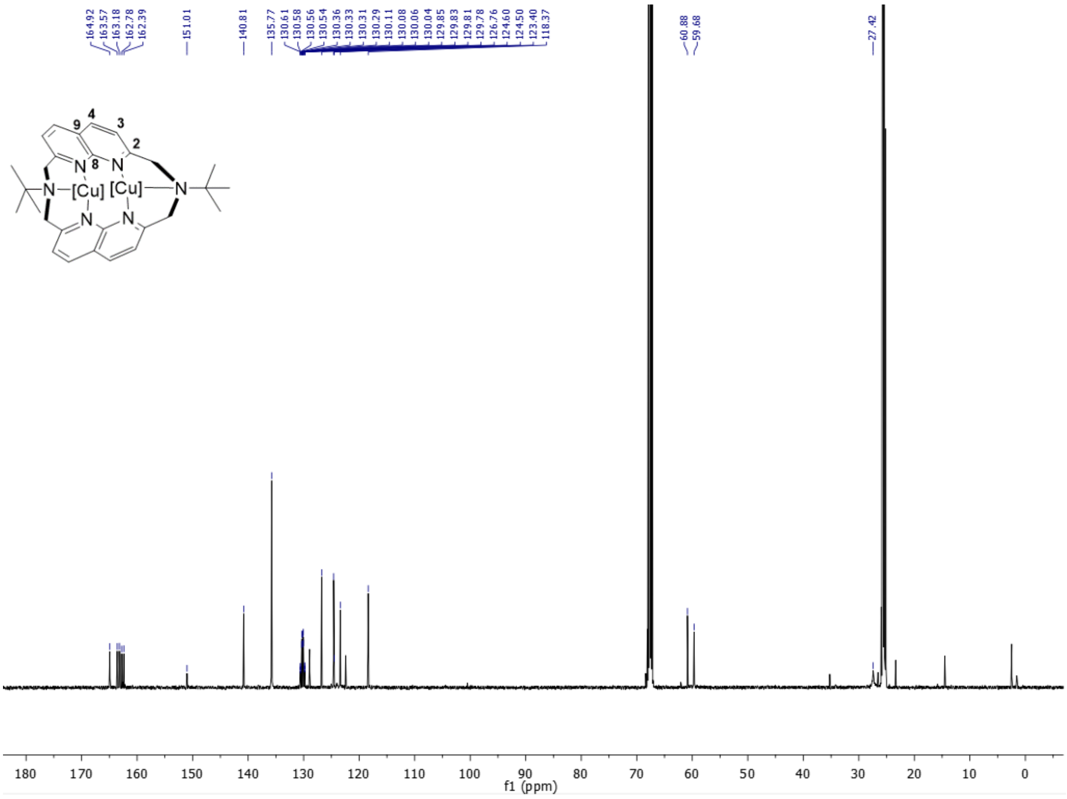

Figure S16. $^{13}C\{^{1}H\}$ NMR spectrum of **1·B(Ar^{F})$_4$** in THF–d_8 at 25 ºC.

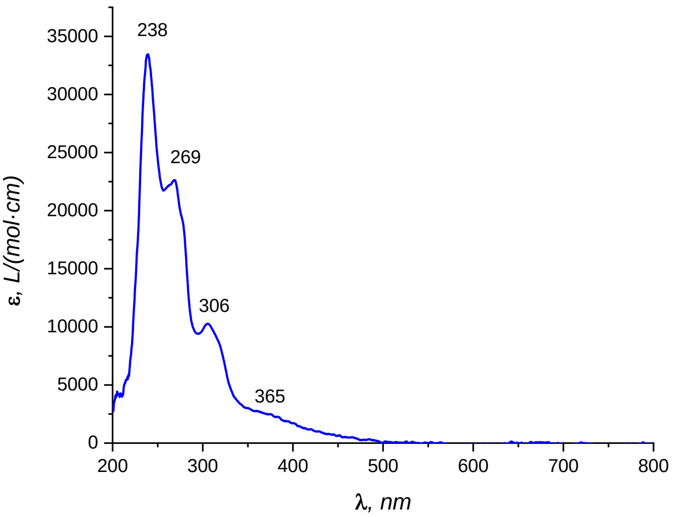

Figure S17. UV–visible spectrum of **1·B(Ar^{F})$_4$** in CH_3CN, the thickness of the quartz cell is 1 cm (90 μM).

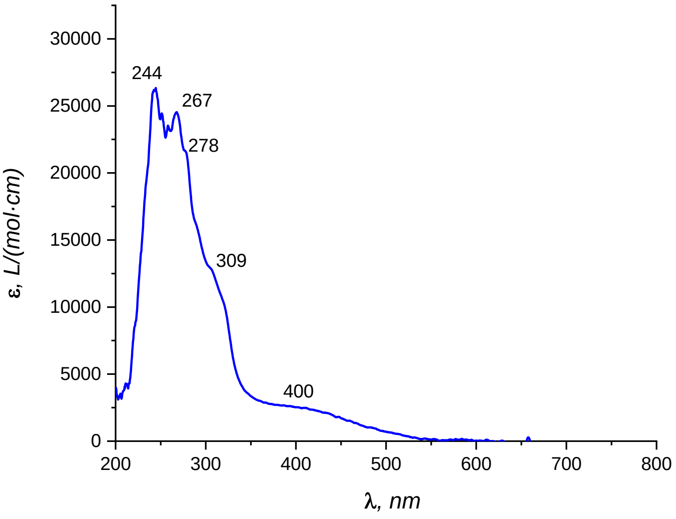

Figure S18. UV–visible spectrum of **1·B(Ar^{F})$_4$** in THF, the thickness of the quartz cell is 1 cm (90 μM).

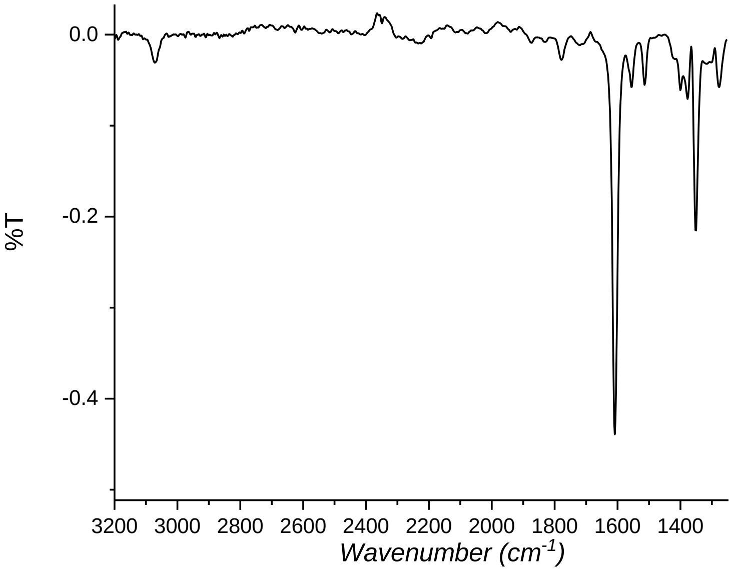

Figure S19. IR spectrum of **1·B(Ar^{F})$_4$** in THF (19.8 mM).

Complex [$Cu_2(^{tBu}N6)_2$][BF_4]$_2$, 2:

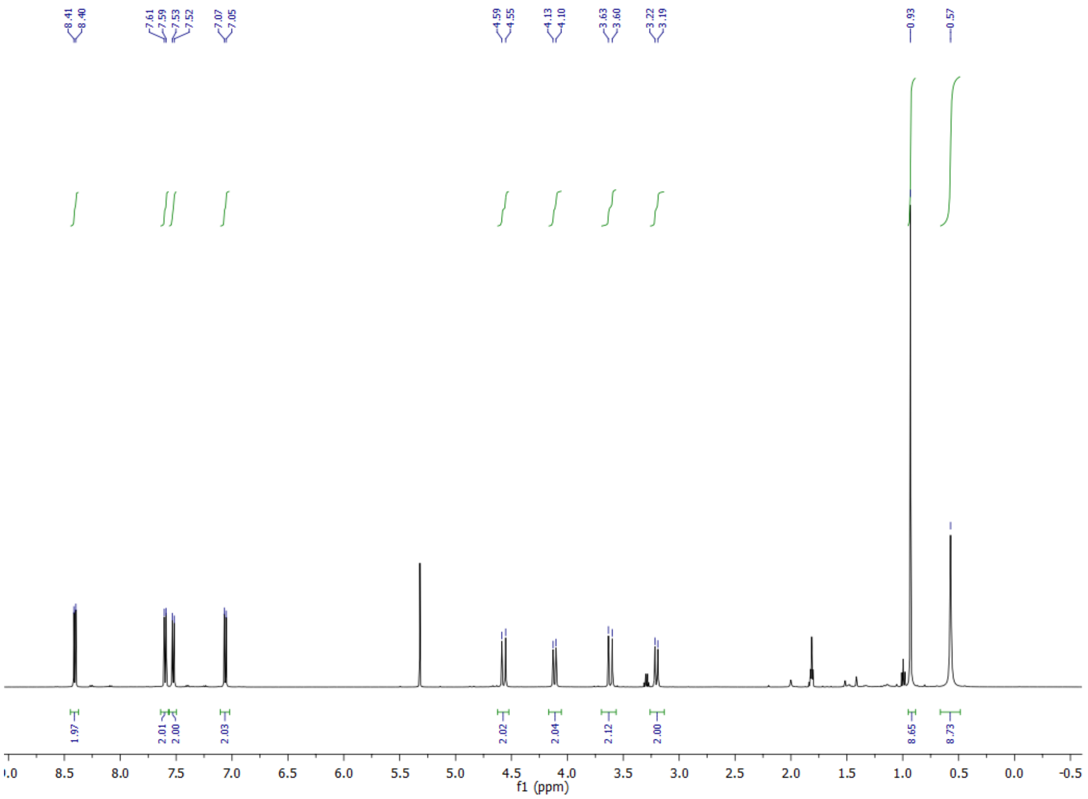

Figure S20. ^{1}H NMR spectrum of **2** in CH_3CN–d_3 at 25 ºC.

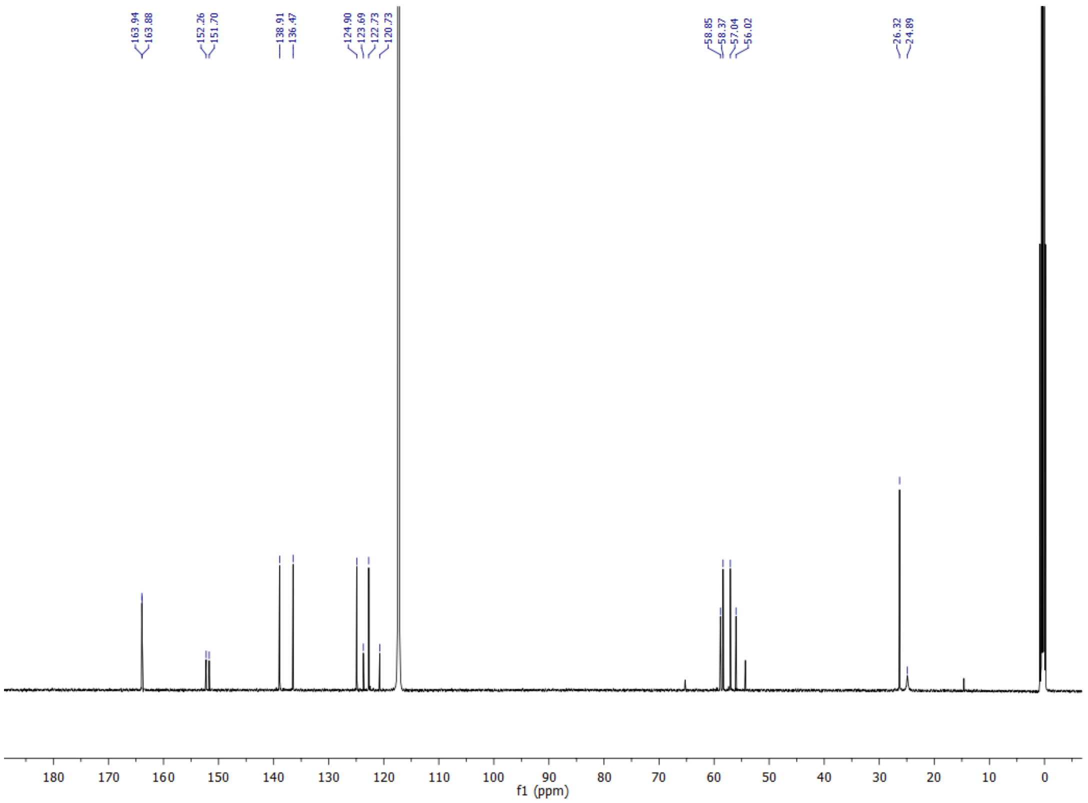

Figure S21. $^{13}C\{^1H\}$ NMR spectrum of **2** in CH_3CN–d_3 at 25 ºC.

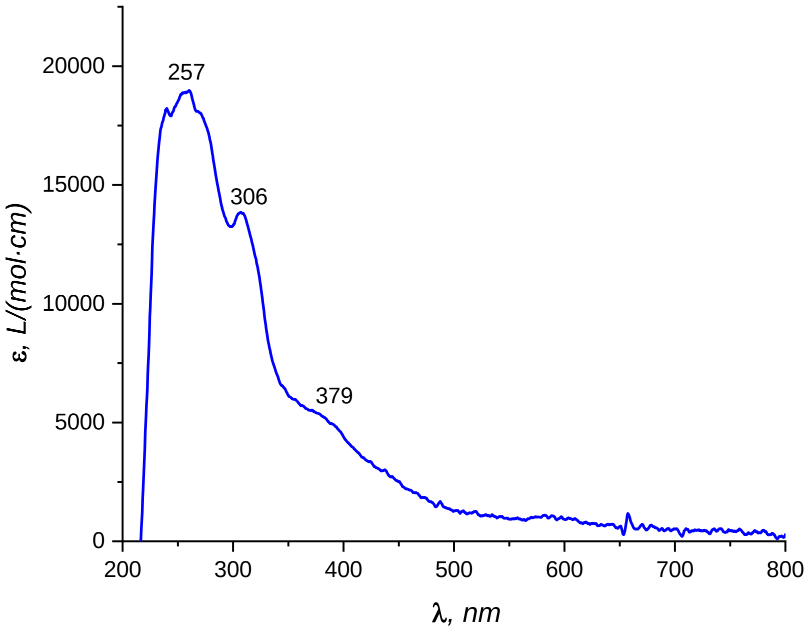

Figure S22. UV–visible spectrum of **2** in CH_3CN, the thickness of the quartz cell is 1 cm (90 µM).

Complex [Cu_2(Cl)^{tBu}N6][BF_4], 3:

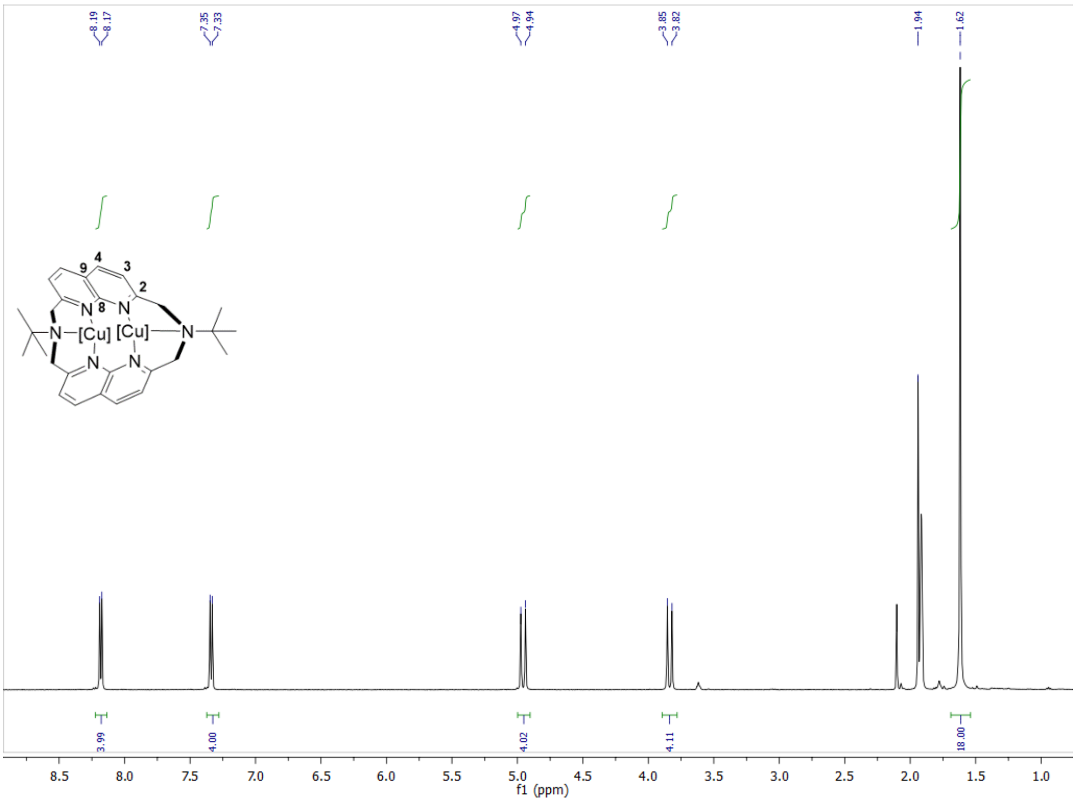

Figure S23. ^{1}H NMR spectrum of **3** in CH_3CN–d_3 at 25 ºC.

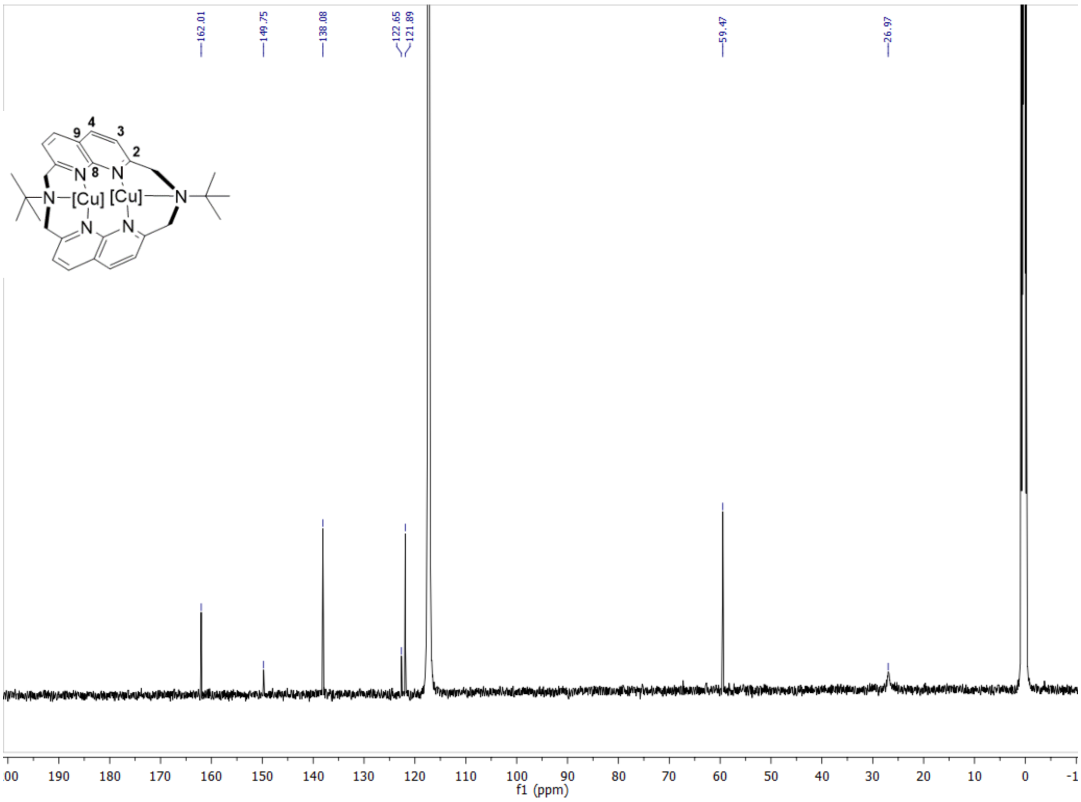

Figure S24. $^{13}C\{^1H\}$ NMR spectrum of **3** in CH_3CN–d_3 at 25 ºC.

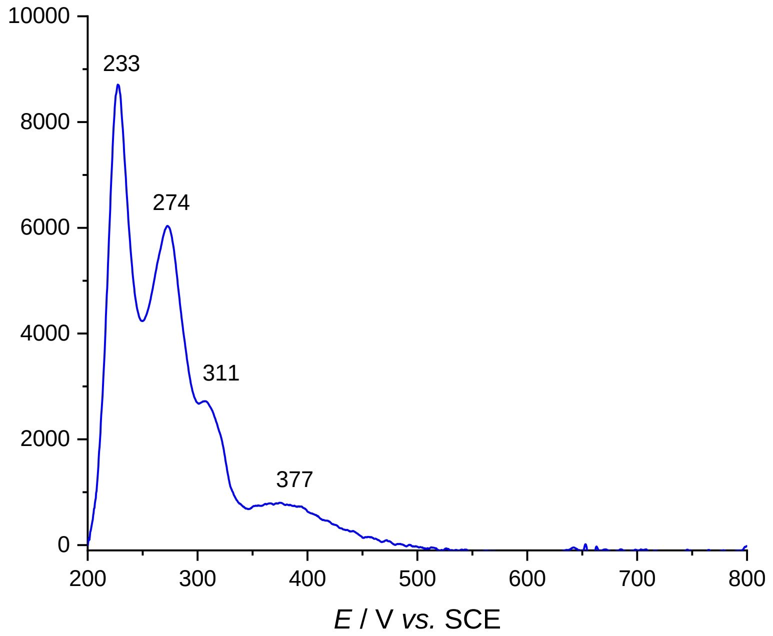

Figure S25. UV–visible spectrum of **3** in CH$_3$CN, the thickness of the quartz cell is 1 cm (90 μM).

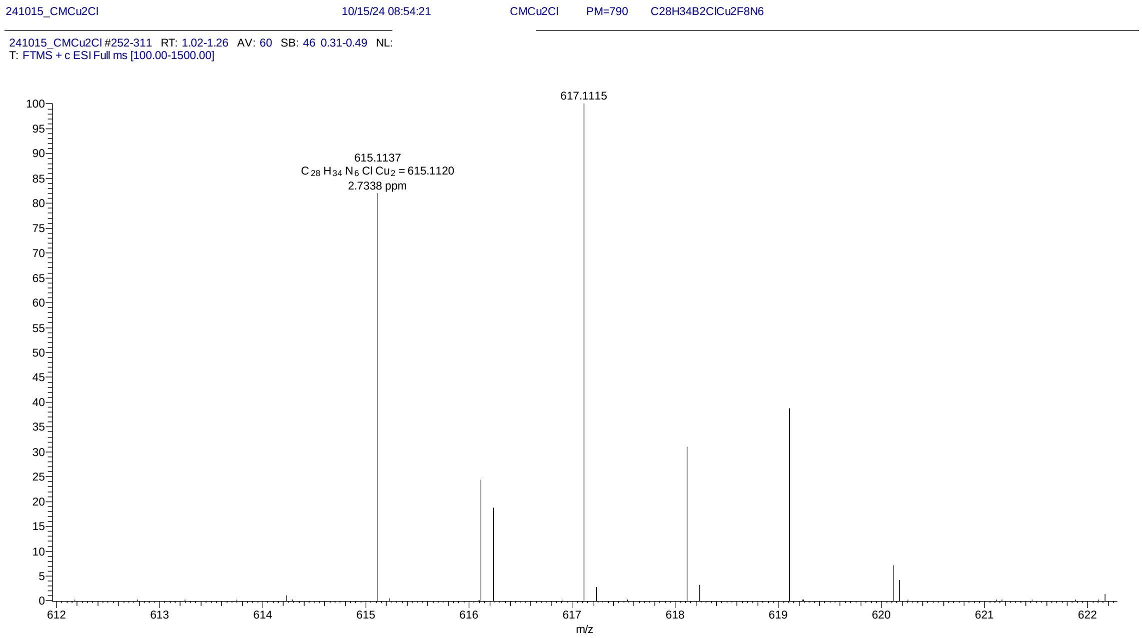

Figure S26. Experimental ESI–MS spectrum of **3**.

Complex $[Cu_2(CO)_2(^{tBu}N6)][B(Ar^F)_4]_2$, 4·$B(Ar^F)_4$:

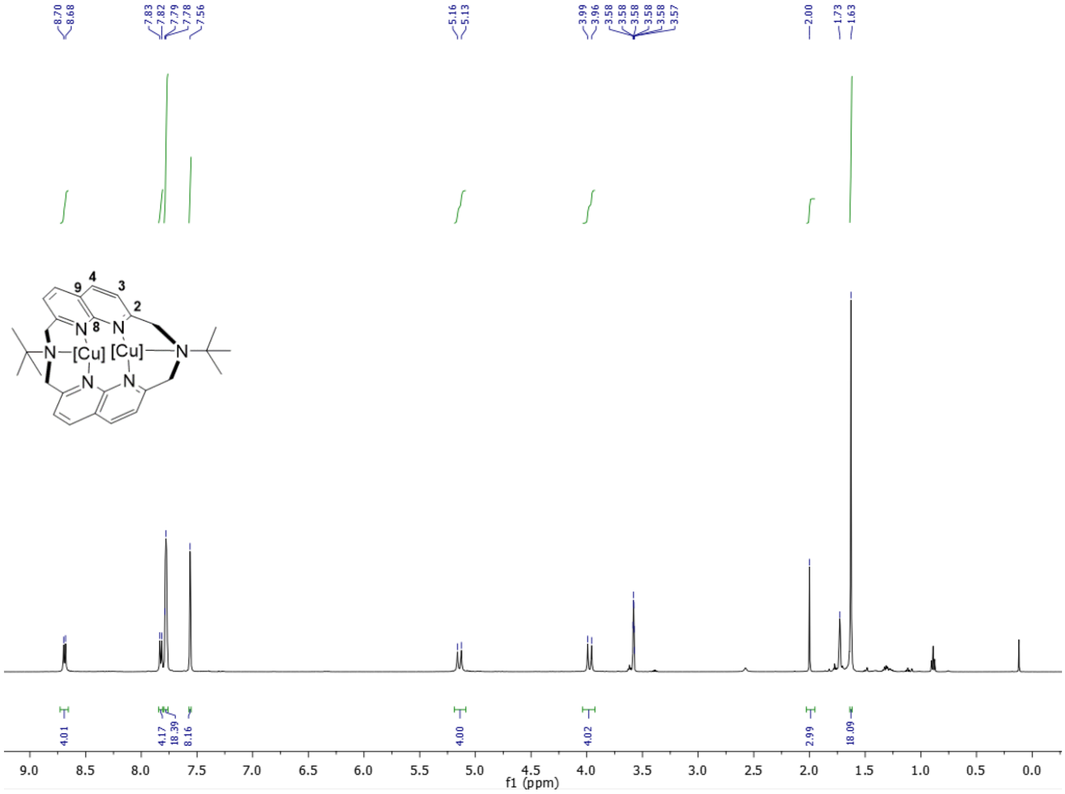

Figure S27. ^{1}H NMR spectrum of **4·$B(Ar^F)_4$** in THF–d_8 at 25 ºC under CO atm.

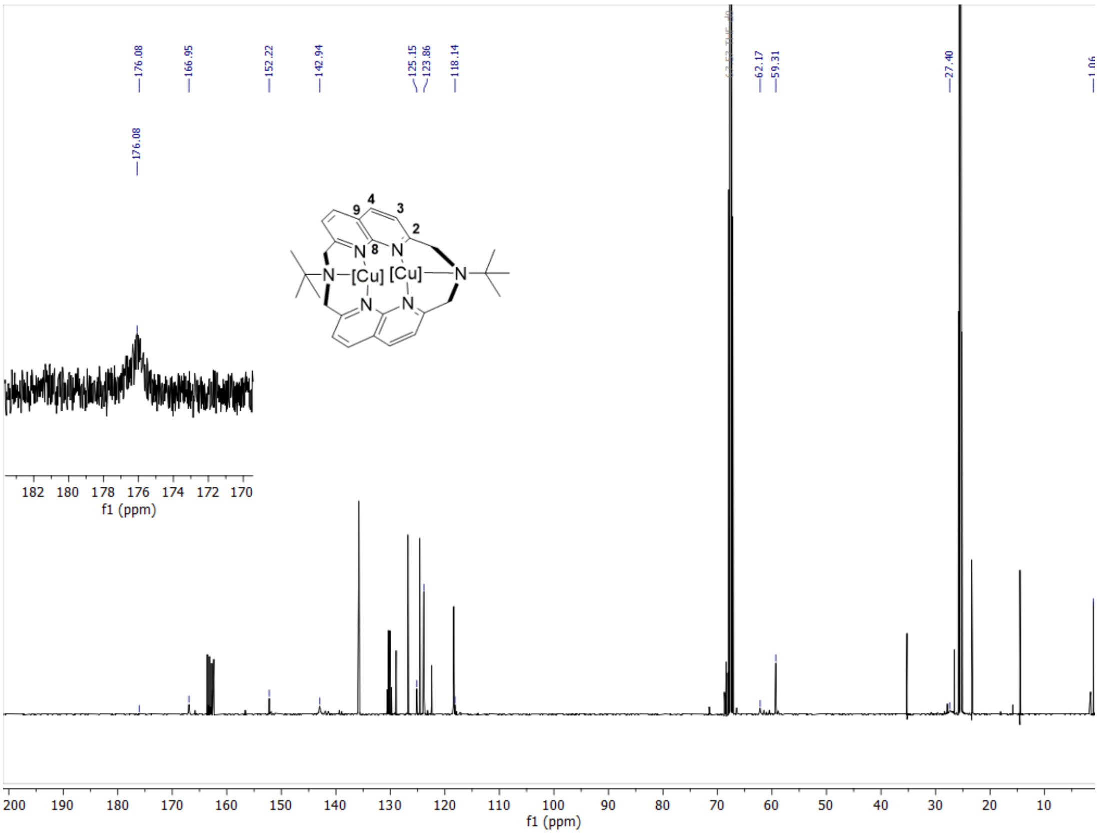

Figure S28. $^{13}C\{^1H\}$ NMR spectrum of **4·B(Ar^F)$_4$** in THF–d_8 at 25 ºC under CO atm.

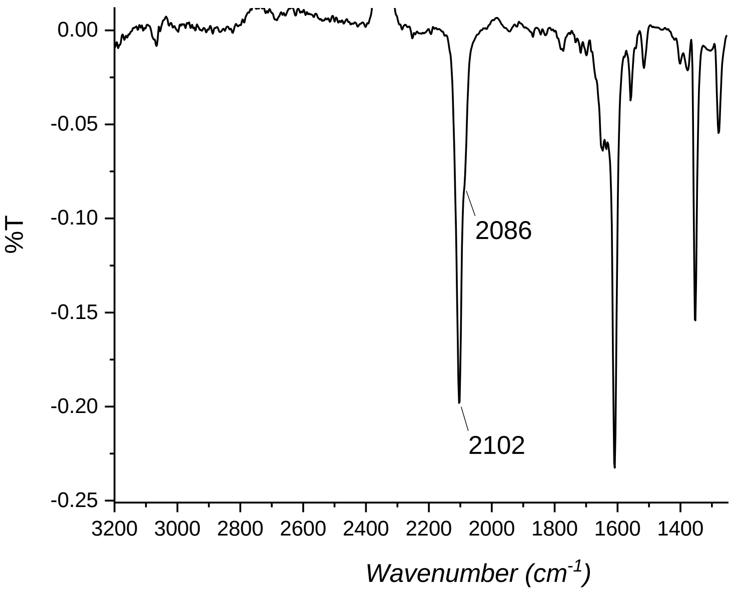

Figure S29. IR spectrum of **4·B(Ar^F)$_4$** in THF (19.8mM) under CO atmosphere.

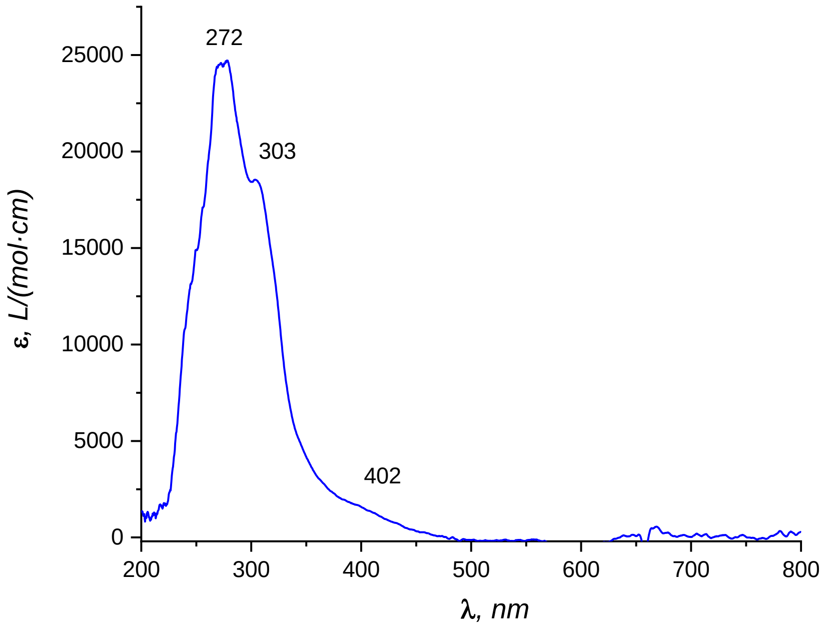

Figure S30. UV–visible spectrum of **4·B(Ar^F)$_4$** in THF under CO atmosphere, the thickness of the quartz cell is 1 cm (90 µM).

When the NMR tube containing complex **4·B(Ar^F)$_4$** under 1 atm. of CO is once again subjected to freeze–pump–thaw and filled with 1 atm. of Ar gas, the solution in the tube did not show any change to the naked eye. The complex formed, exhibit the NMR signals shown in the figures S25 and S26.

We have attempted in several occasions the isolation of complex **4·B(Ar^F)$_4$** in the solid state and/or via crystallization. However, all our attempts were not fruitful. Furthermore, Complex **4·B(Ar^F)$_4$** is unstable in solution under Ar atmosphere, showing decomposition withing 48h, observing the formation of black precipitate in the bottom of the NMR tube.

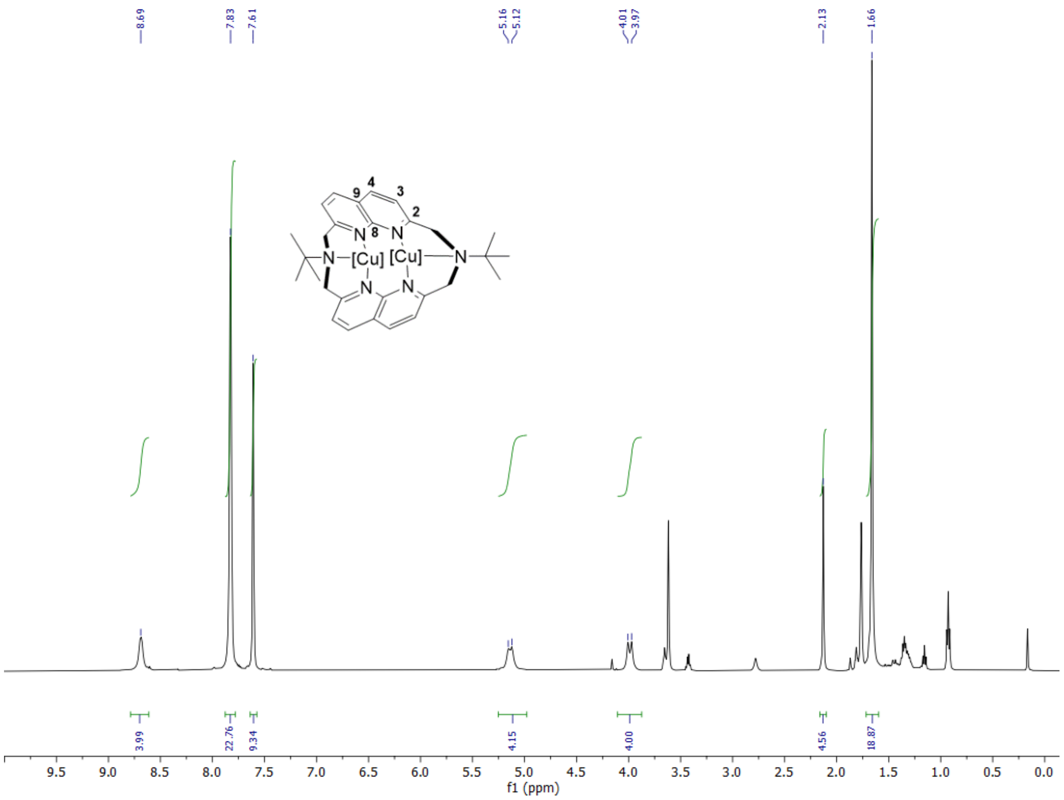

Figure S31. ^{1}H NMR spectrum of **4·B(Ar^{F})$_4$** in THF–d_8 at 25 ºC under Ar atm.

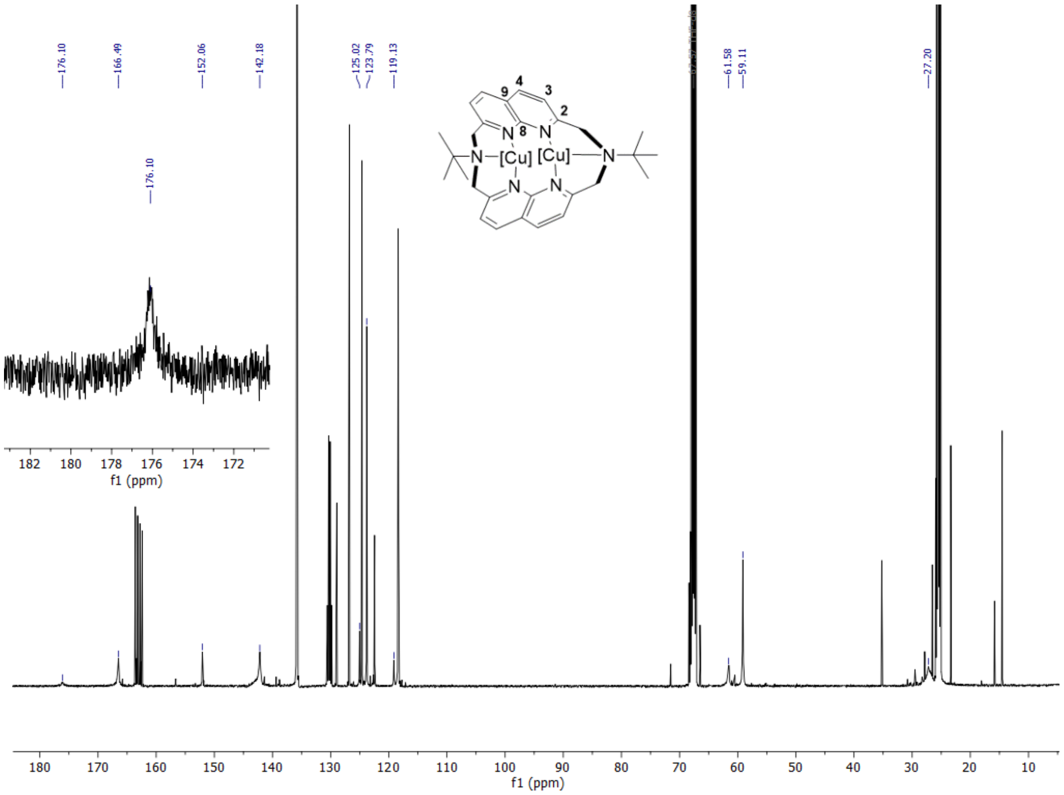

Figure S32. $^{13}C\{^{1}H\}$ NMR spectrum of **4·B(Ar^{F})$_4$** in THF–d_8 at 25 ºC under Ar atm.

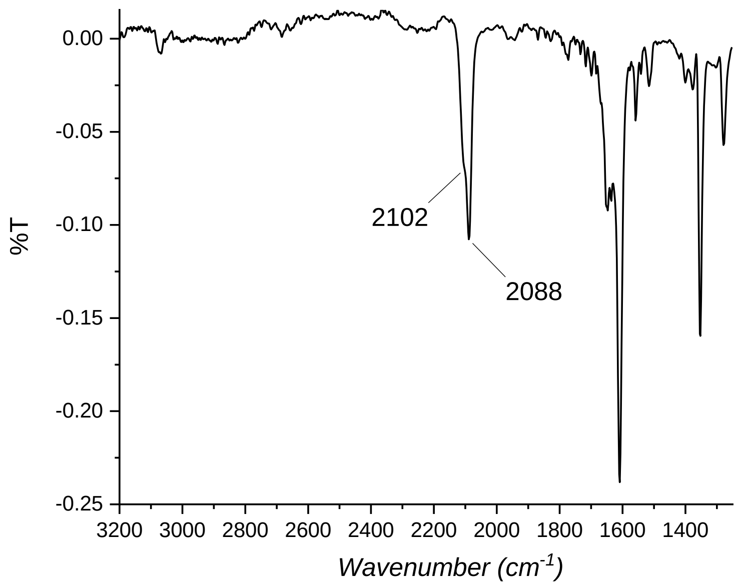

Figure S33. IR spectrum of **4·B(Ar^{F})$_4$** in THF (19.8mM) under Ar atmosphere.

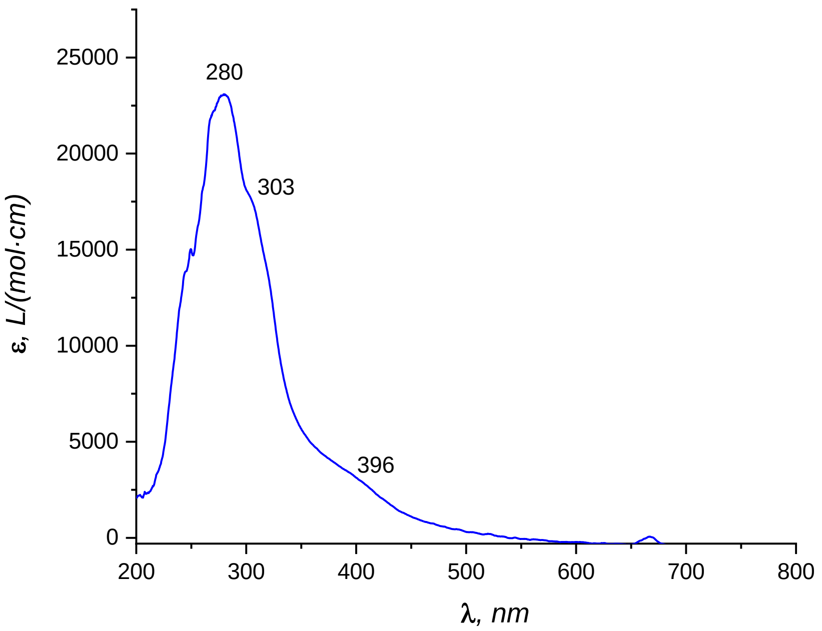

Figure S34. UV–visible spectrum of **4·B(Ar^{F})$_4$** in THF under Ar atmosphere, the thickness of the quartz cell is 1 cm (90 μM).

Synthesis of complex **4·B(Ar^F)$_4$**, without MeCN in solution. Inside the glovebox, complex **1·B(Ar^F)$_4$** (0.04 g, 0.017 mmol) is dissolved in 1 ml of dry 1,2-dimethoxyethane and transferred to an ampule. Once outside, the solvent is removed by bubbling a stream of CO till the ampule is dry. This process is repeated 3 times. Later the solid obtained is dissolved in THF-d_8 and the solution transferred to a Heavy Wall Quick Pressure Valve NMR Tube (Wilmad) under CO. The NRM spectra for this complex are shown in Figures S35 and S34. Complex **4·B(Ar^F)$_4$** obtained under these experimental conditions exhibits the same set of NMR signals as shown as in Figures S27 and S28.

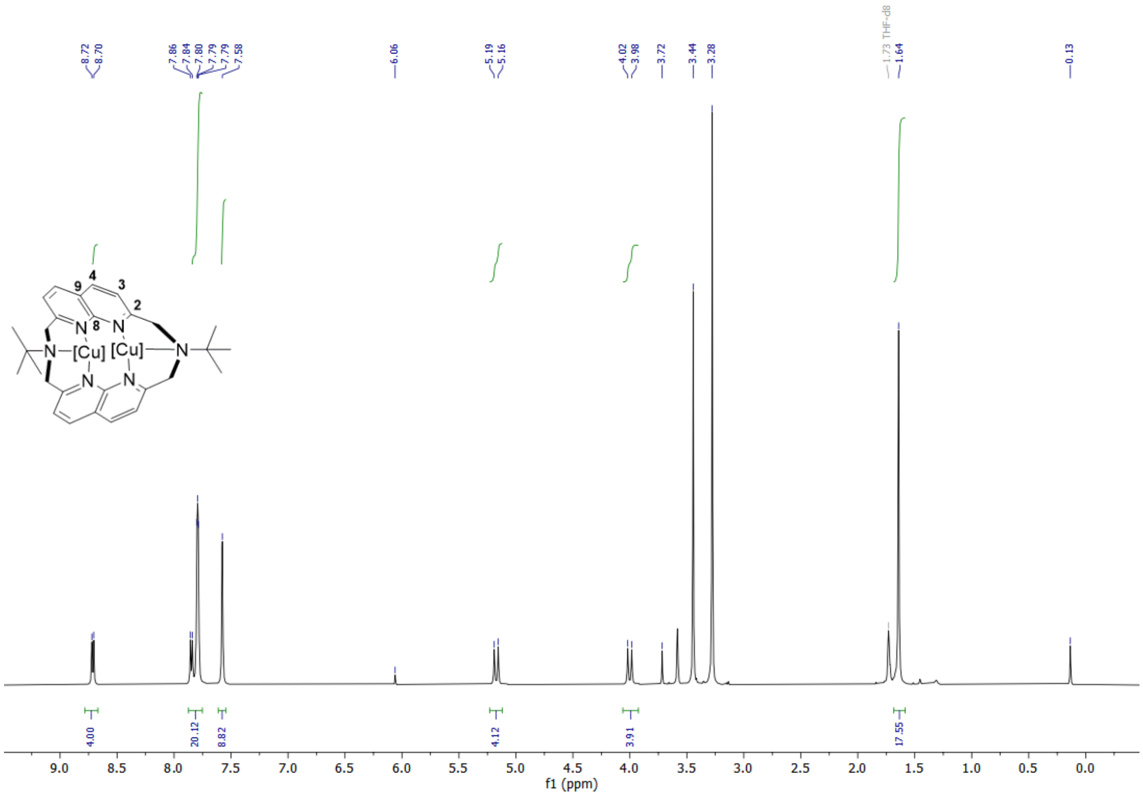

Figure S35. ^{1}H NMR spectrum of **4·B(Ar^F)$_4$** in THF–d_8 at 25 ºC under CO atm. Singlets at 6.06 ppm and 3.72 ppm belongs to internal standard 1,3,5-trimethoxybenzene and singlets at 3.44 ppm and 3.28 ppm belongs to 1,2-dimethoxyethane. No singlet for MeCN is observed.

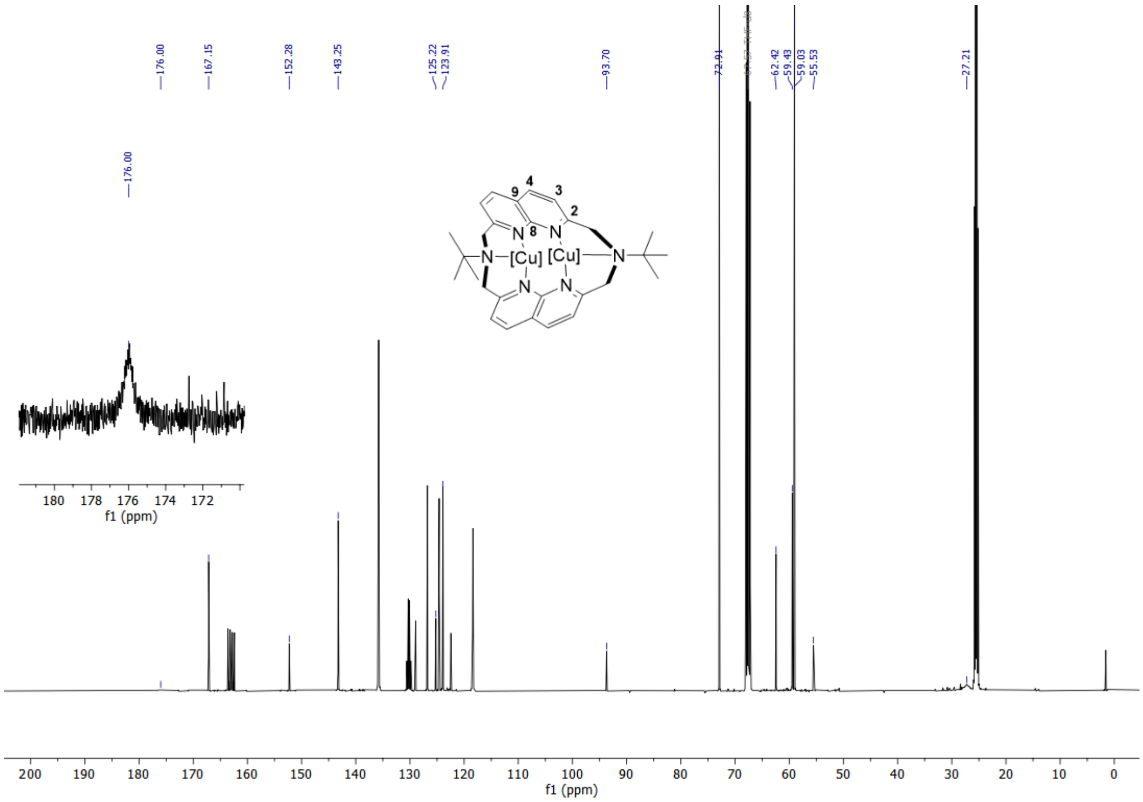

Figure S36. ^{13}C{^{1}H} NMR spectrum of **4·B(Ar^{F})$_{4}$** in THF–d_8 at 25 ºC under CO atm. Singlets at 93.7 ppm and 53.5 ppm belongs to internal standard 1,3,5-trimethoxybenzene and singlets at 72.9 ppm and 59.0 ppm belongs to 1,2-dimethoxyethane.

When the NMR tube containing complex **4·B(Ar^{F})$_{4}$** under 1 atm. of CO is subjected to freeze–pump–thaw (4 times) and filled with 1 atm. of Ar gas, the complex formed exhibits the NMR signals shown in the figures S37 and S38. Complex **4·B(Ar^{F})$_{4}$** obtained under these experimental conditions exhibits the same set of NMR signals as shown as in Figures S31 and S32, however in this occasion the signals are less broad.

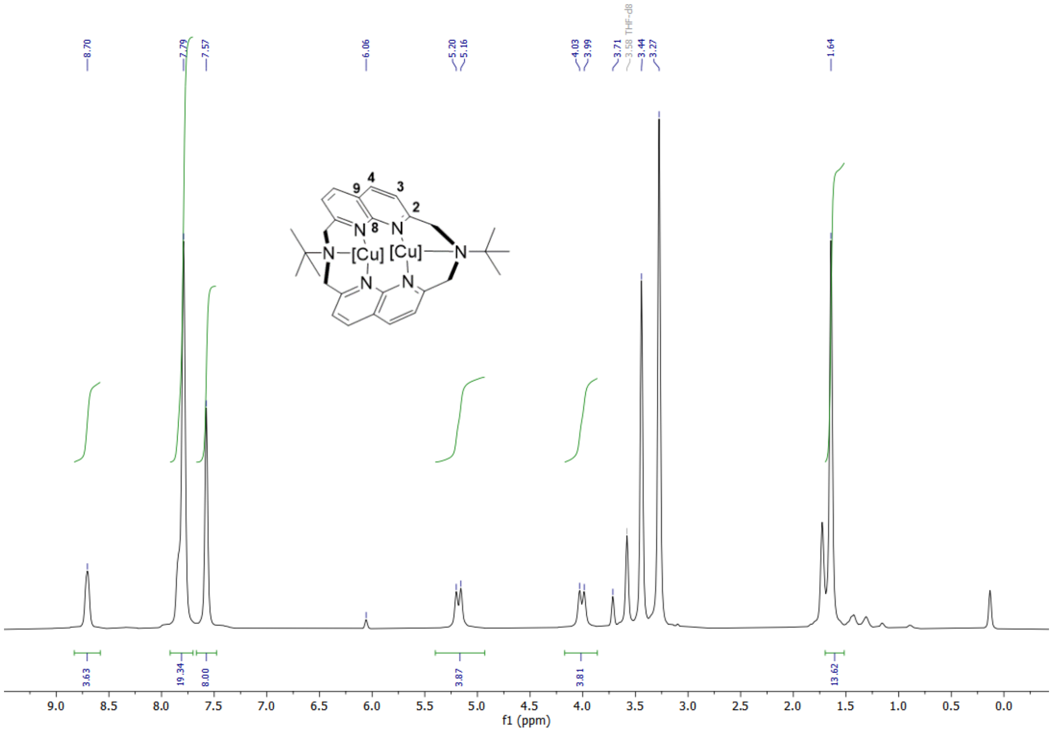

Figure S37. ^{1}H NMR spectrum of **4·B(Ar^{F})$_4$** in THF–d_8 at 25 ºC under Ar atm. 1,3,5-trimethoxybenzene (6.06 and 3.71 ppm), DME (3.44 and 3.27 ppm). No MeCN is observed.

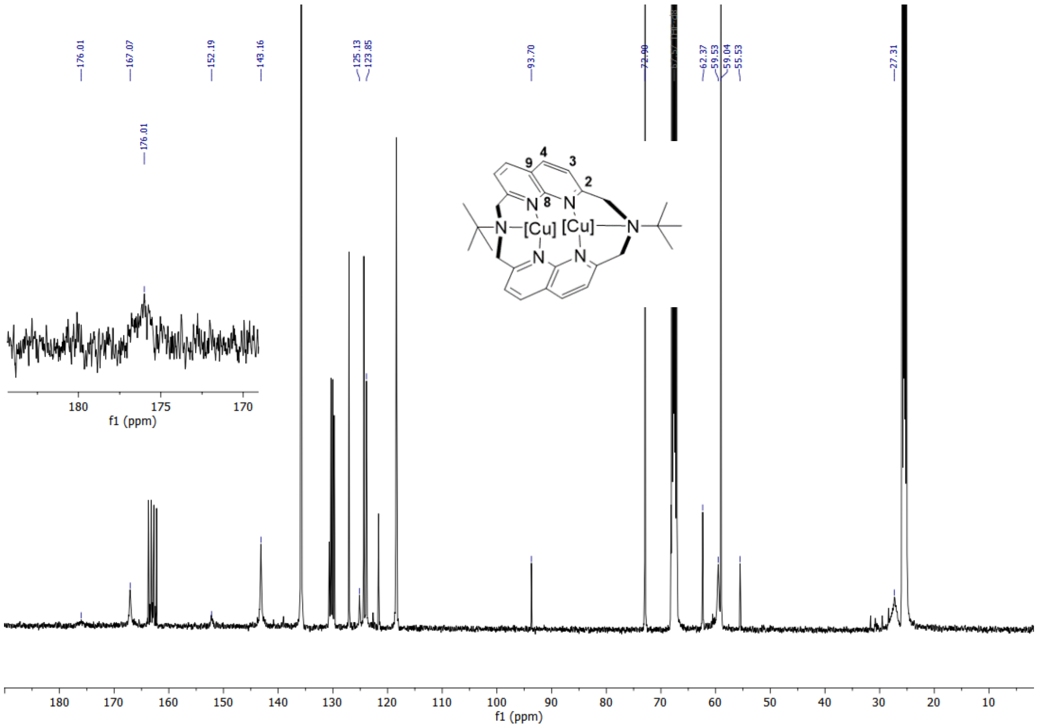

Figure S38. $^{13}C\{^{1}H\}$ NMR spectrum of **4·B(Ar^{F})$_4$** in THF–d_8 at 25 ºC under Ar atm. 1,3,5-trimethoxybenzene (93.7 and 53.5 ppm), DME (72.9 and 59.0 ppm). No MeCN is observed.

Distribution between 1·BF_4 and 2 in acetonitrile solutions

7 mg of complex **1·BF_4** were dissolved in 0.4 mL of CH_3CN-d_3 inside the glovebox and transferred to a screw cap NMR tube. The solution was analyzed by ^{1}H NMR, performing each measurement at a specific temperature with increments of 10º C, from 20º to 60º C, and waiting 15 min after each temperature was stabilized. These measurements were performed four times. The percentual ratios obtained from measuring the integrals of **1·BF_4** (signal from 7.35 to 7.52 ppm) and of **2** (signals from 7.53 to 7.72 ppm) are shown below in Table S1, along with the standard deviation of these measurements. 1,3,5-Trimethoxybenzene was used as an internal standard.

Table S1. Ratios obtained from recording 1H NMR spectra of complex **1·BF_4** at different temperatures.

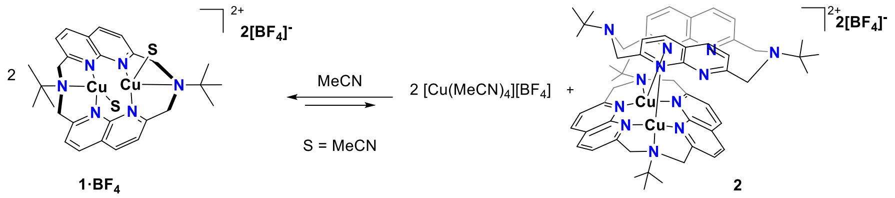

T (º C)	2 (%)	1·BF_4 (%)	Std. Dev.
20	10,8	89,2	1,1
30	12,5	87,5	1,7
40	14,2	85,8	1,9
50	15,7	84,3	2,0
60	17,5	82,5	2,4

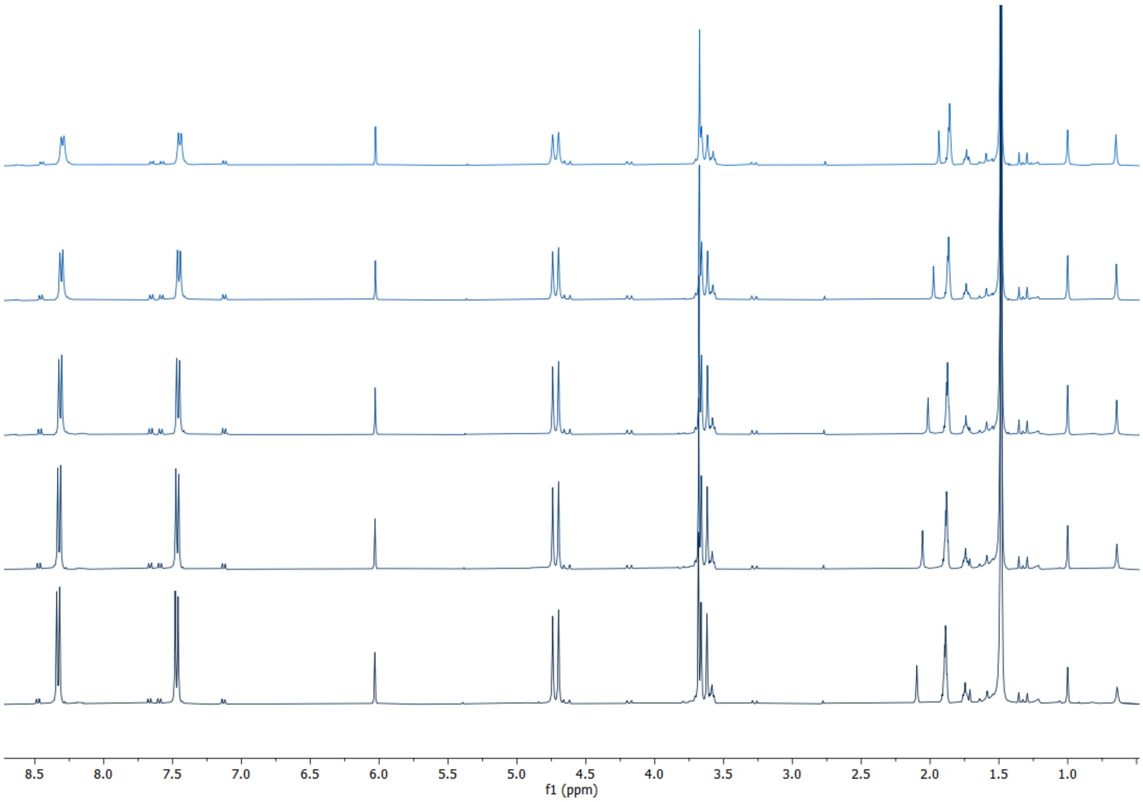

Figure S39. ^{1}H NMRs spectra of **1·BF₄** in CH_3CN-d_3 at different temperatures ranging from 20º (bottom) to 60º C (top) with a 10º C difference, under Ar atm. Singlets at 6 ppm and 3.6 ppm belong to 1,3,5-Trimethoxybenzene (internal standard).

Computational Details

Density functional theory (DFT) calculations were performed in ORCA 5.0.3[1] without geometry constraints, using the PBE0 functional[2] and the double–ζ def2–SVP basis set[3] for all atoms (basis set 1, BS1). Dispersion effects were included by applying the D3 version of Grimme's dispersion correction with the Becke–Johnson damping function (D3BJ)[4]. Bulk solvent effects were included using the CPCM model. Calculations were carried out without the RIJCOSX approximation (the keyword NORI was employed), as previous experience revealed that this approximation led to small imaginary frequencies difficult to remove. Vibrational analysis was performed at the same level of theory on the optimized geometries to characterize them as true minima in the potential energy surface by the absence of imaginary frequencies. All energies in solution were corrected by single-point calculations with a larger basis set 2 (BS2) including triple-ζ def2TZVP basis set for the H, C, N, O atoms and quadruple-ζ def2QZVP for Cu.[3] In order to obtain accurate values of the vibration frequencies, a scaling factor of 0.9592 was included in the calculations using BS1, as reported by Wilson *et al.*[5] for high vibrational (> 1000 cm^{-1}) frequencies using the PBE0 functional and a double-zeta basis set. Visualization of the calculated structures and spectra was performed using Chemcraft.[6] QTAIM analysis was carried out using Multiwfn software[7] on electron densities of the optimized structures.

Complex 4_bridging CO

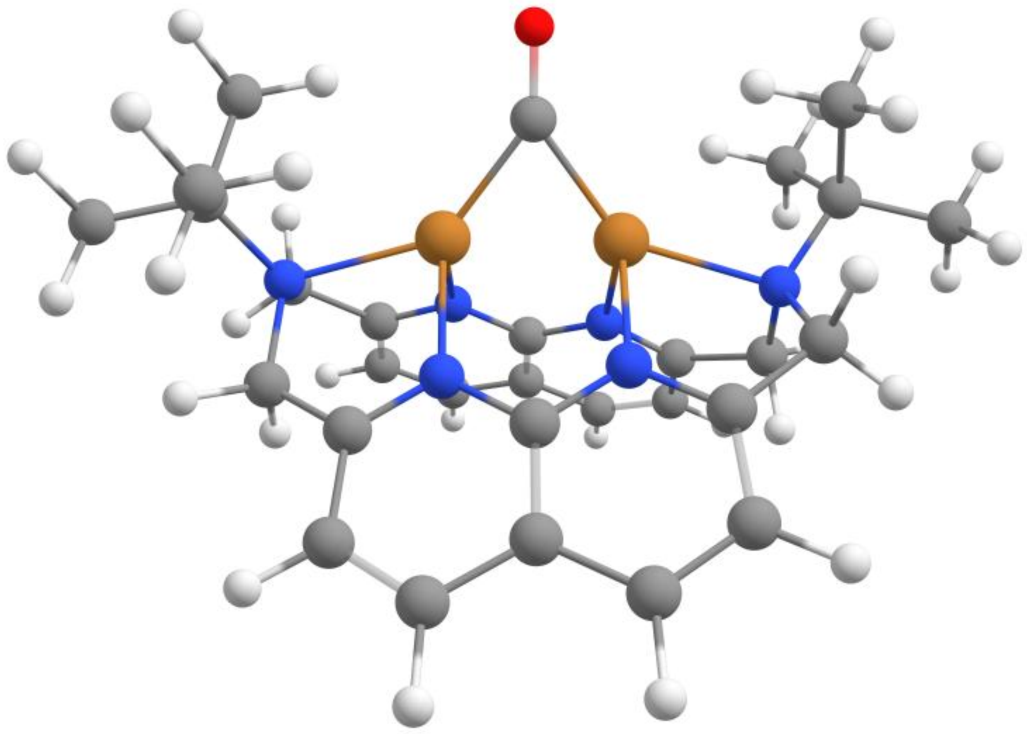

Figure S40. Calculated molecular geometry of the cationic fragment of **4_bridgingCO**.

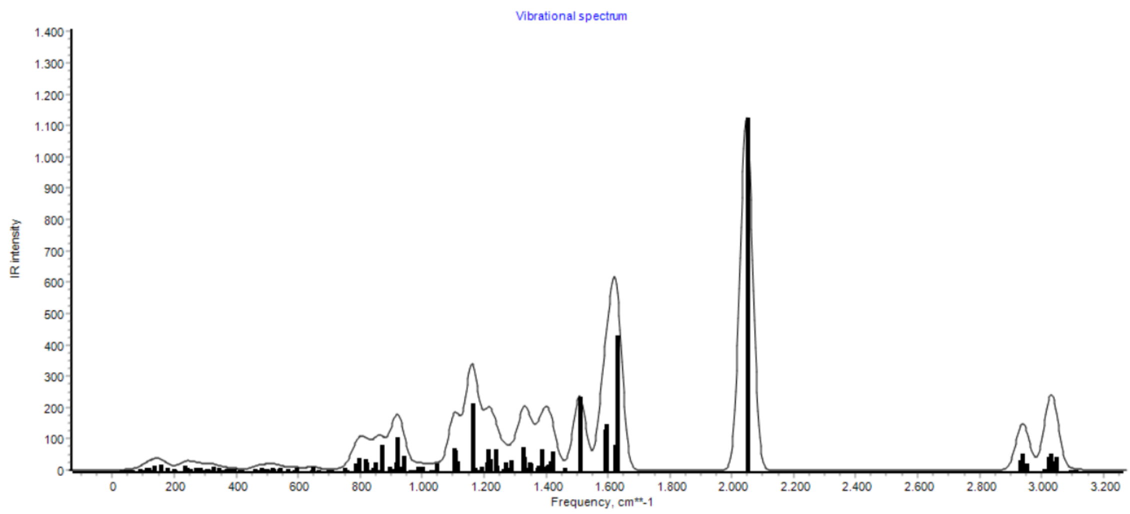

Figure S41. Computed vibrational spectrum of **4_bridgingCO**, using a Gaussian broadening for the predicted bands.

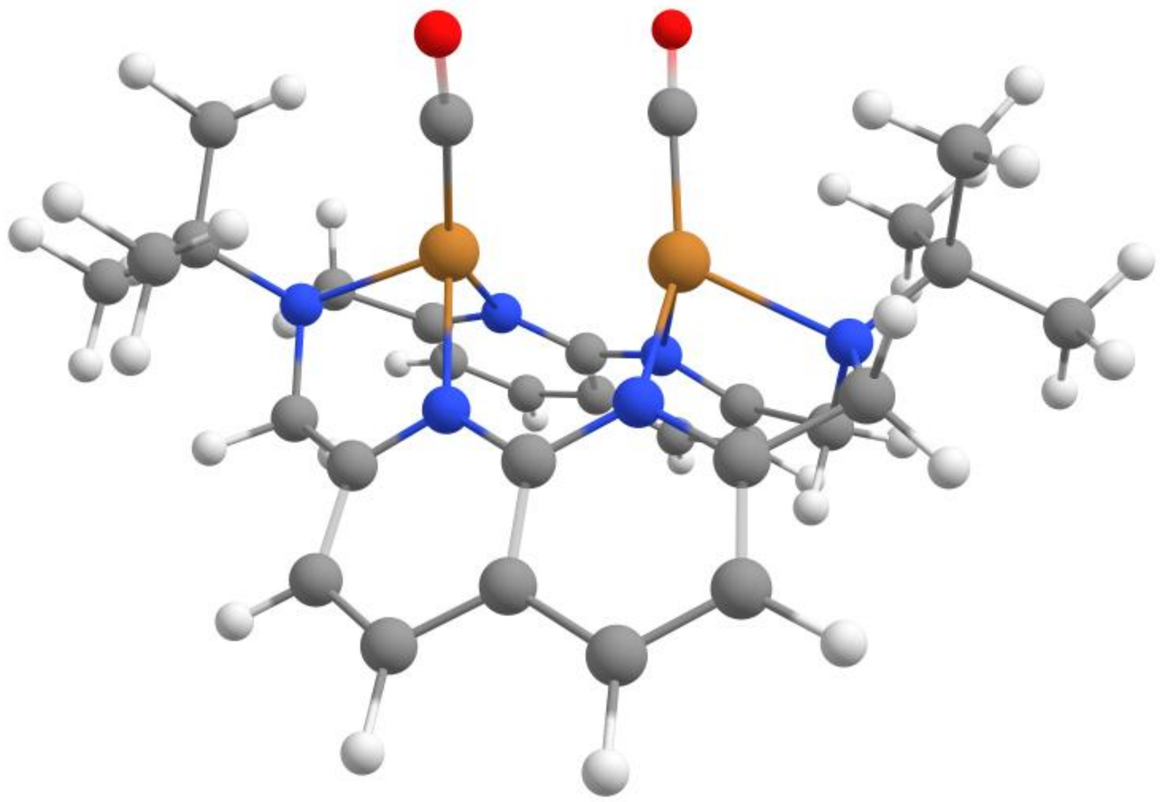

Figure S42. Calculated molecular geometry of the cationic fragment of **4·B(Ar^{F})$_4$**.

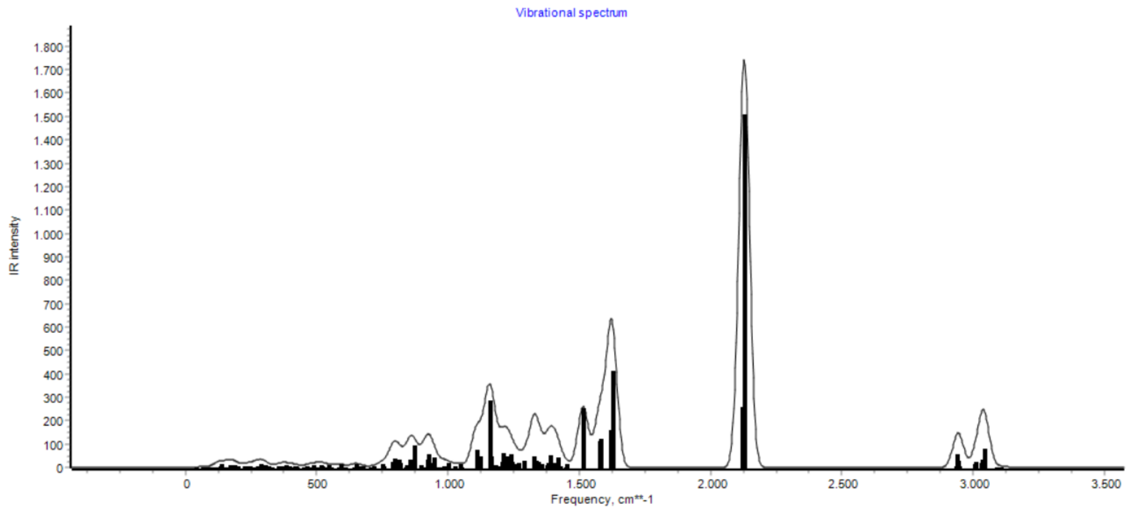

Figure S43. Computed vibrational spectrum of **4·B(Ar^{F})$_4$**, using a Gaussian broadening for the predicted bands.

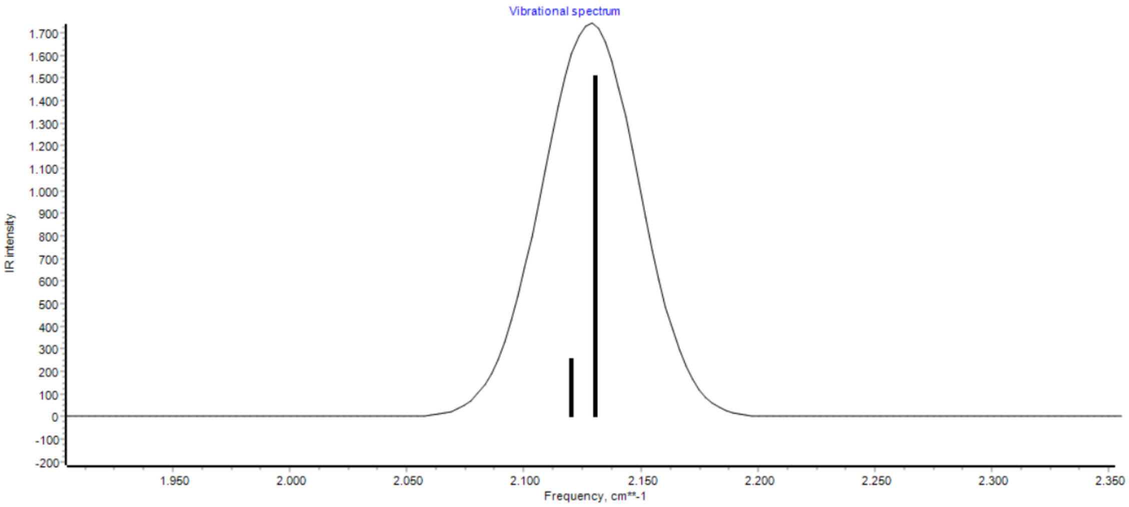

Figure S44. Zoom–in of the CO region in the computed vibrational spectrum of **4·B(Ar^{F})$_4$**.

Complex Cu(MeCN)Cu(CO)

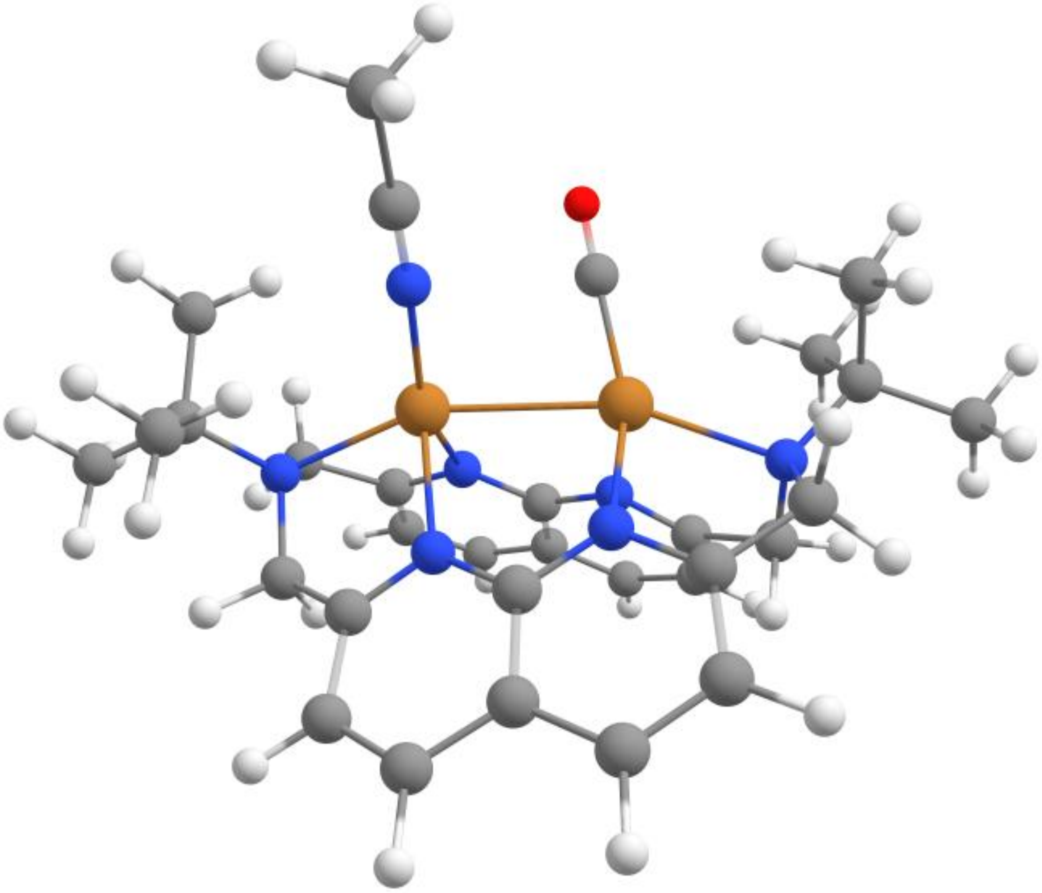

Figure S45. Calculated molecular geometry of the cationic fragment of **Cu(MeCN)Cu(CO)**.

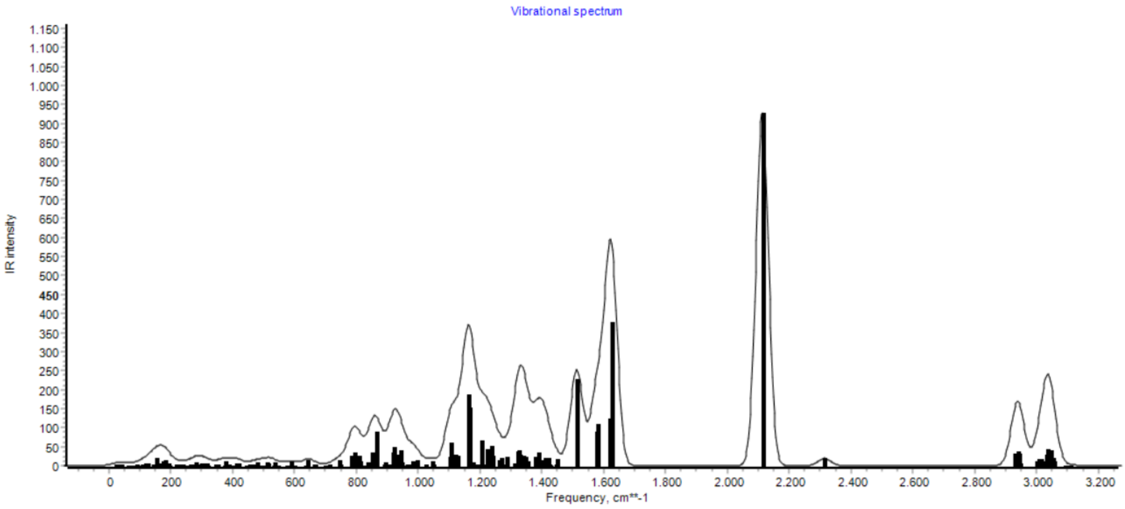

Figure S46. Computed vibrational spectrum of **Cu(MeCN)Cu(CO)**, using a Gaussian broadening for the predicted bands.

- **Equilibrium between complex 1 and CO**

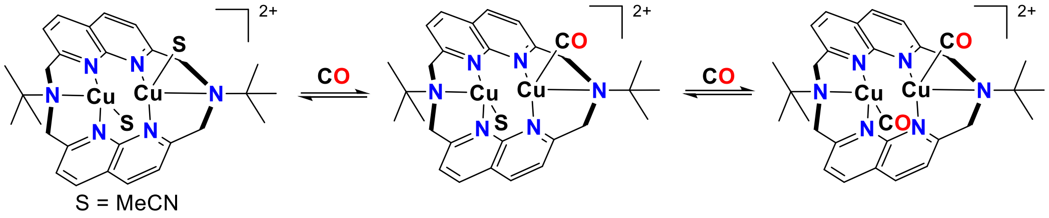

1 **Cu(MeCN)Cu(CO)** **4·B(ArF)4**

-6.7 -10.9

Considering complex **1** + CO as the energy reference, the formation of **Cu(MeCN)Cu(CO)** is exergonic (ΔG = -6.7 kcal mol^{-1}). However, displacement of the bound MeCN by another CO equivalent gives complex **4·B(Ar^{F})$_4$**, which is thermodynamically favorable (ΔG = -10.9 kcal mol^{-1}). Therefore, these data indicate that species **4·B(Ar^{F})$_4$** is more stable than **Cu(MeCN)Cu(CO)**. Taking this energy difference (ΔG = -4.2 kcal mol^{-1}) in the $\Delta G = -RT\ln Keq$ equation gives a K_{eq} = 1200, which suggests that the equilibrium is strongly shifted to the formation of **4·B(Ar^{F})$_4$**.

Given that in THF there seems to be just one bridging MeCN molecule, the aforementioned equilibrium was considered, using **Cu(MeCN)Cu** as starting point instead of **1**. Similar results were obtained, as **4·B(Ar^{F})$_4$** is more stable than **Cu(MeCN)Cu(CO)** by 4.3 kcal mol^{-1}. Using this energy difference, K_{eq} = 1425, which again supports that the equlibrium is strongly shifted to the formation of **4·B(Ar^{F})$_4$**.

- **QTAIM analysis**

In order to investigate potential cuprophilic interactions in the synthesized complexes, QTAIM analysis was performed on the optimized structures. Bond critical points (BCPs)

between the Cu centers are observed for all the complexes considered, as depicted in the representation of the laplacian of the electron density:

Complex **1·X**

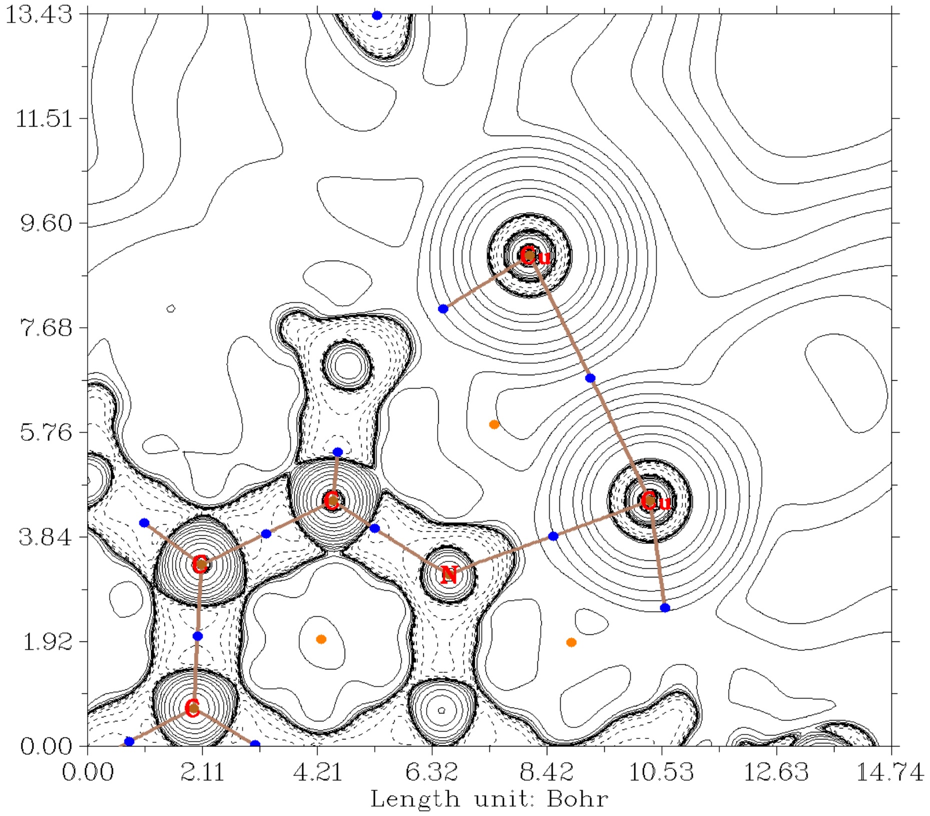

Figure S47. Plot of the Laplacian ($\nabla^2\rho$) of the electronic density of complex **1·X**.

Complex **2**

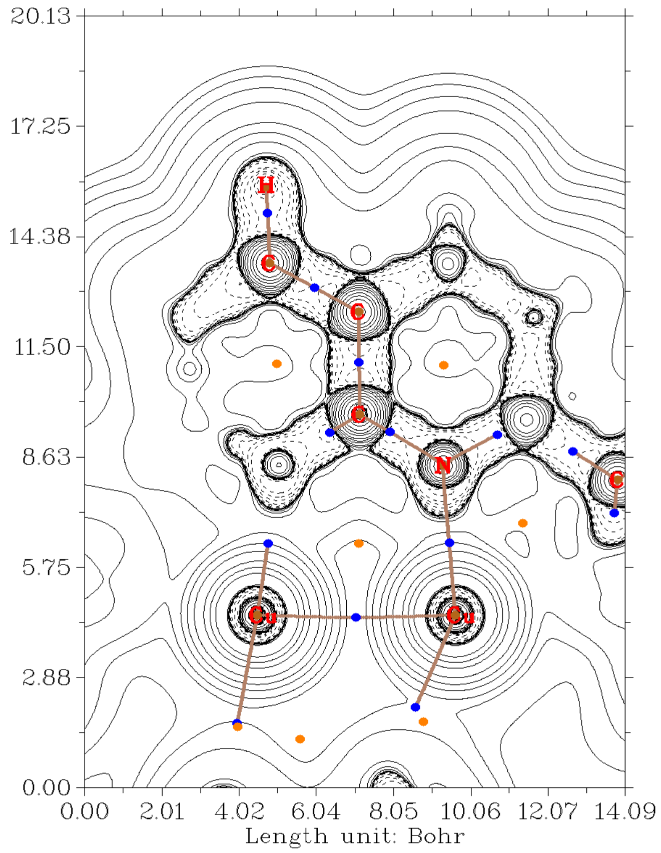

Figure S48. Plot of the Laplacian ($\nabla^2\rho$) of the electronic density of complex **2**.

Complex **3**

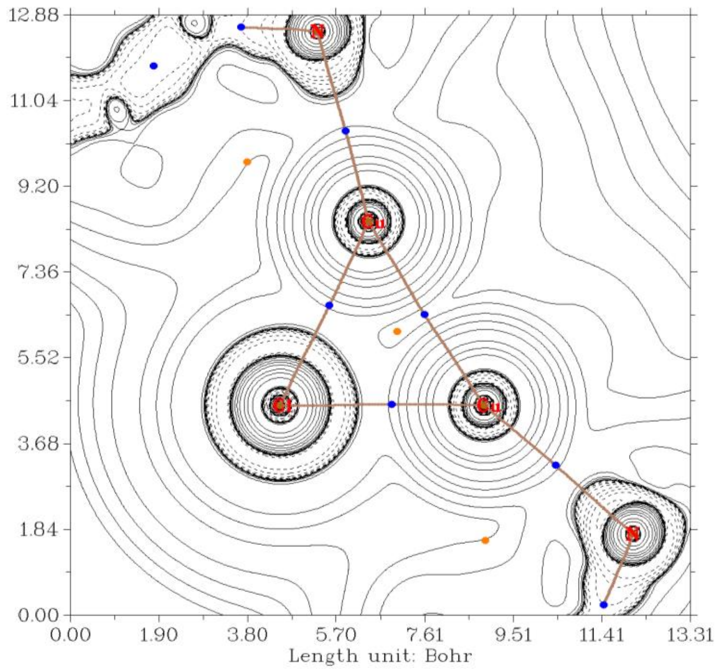

Figure S49. Plot of the Laplacian ($\nabla^2\rho$) of the electronic density of complex **3**.

Complex **4·B(Ar^{F})$_4$**

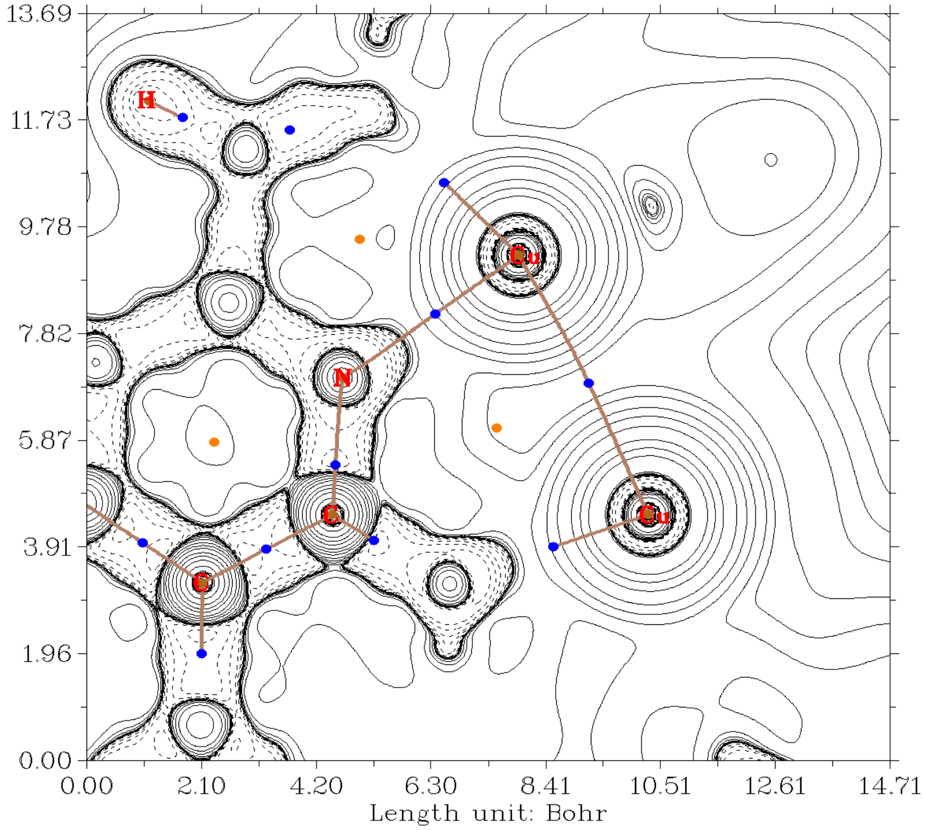

Figure S50. Plot of the Laplacian ($\nabla^2\rho$) of the electronic density of complex **4·B(Ar^{F})$_4$**.

As depicted above, all complexes exhibit bond critical points (BCPs) between the metal centers, which agrees with the presence of potential cuprophilic interactions. The electron density of such BCPs is summarized in the following table:

Complex	Cu···Cu distance (Å)	ρ_{BCP} (a.u.)
**1·X**	2.65	0.028
**2**	2.73	0.025
**3**	2.46	0.040
**4·B(Ar^{F})$_4$**	2.81	0.022

These values are in good agreement with closed-shell metal-metal interactions, similar to those described in *Chem. Eur. J.* **2022**, e202201639 and *Inorg. Chem.* **2022**, *61*, 19333 for other naphthyridine-containing systems.

References from Computational Details

[1] Neese, F.; Wennmohs, F.; Becker, U.; Riplinger, C. The ORCA Quantum Chemistry Program Package. *J. Chem. Phys.* **2020**, *152*, 224108

[2] Adamo, C.; Barone, V. Toward reliable density functional methods without adjustable parameters: The PBE0 model. *J. Chem. Phys.,* **1999**, *110*, 6158–6170

[3] Weigend, F., Ahlrichs, R. Balanced Basis Sets of Split Valence, Triple Zeta Valence and Quadruple Zeta Valence Quality for H to Rn: Design and Assessment of Accuracy. *Phys. Chem. Chem. Phys.* **2005**, *7*, 3297.

[4] a) Grimme, S., Ehrlich, S., Goerigk, L. Effect of the damping function in dispersion corrected density functional theory. *J. Comput. Chem.* **2011**, *32*, 1456; (b) Grimme, S., Antony, J., Ehrlich, S., Krieg, H. A consistent and accurate ab initio parametrization of density functional dispersion correction (DFT–D) for the 94 elements H–Pu. *J. Chem. Phys.* **2010**, *132*, 154104.

[5] Laury, M. L., Carlson, M. J., Wilson, A. K. Vibrational frequency scale factors for density functional theory and the polarization consistent basis sets. *J. Comput. Chem.*, **2012**, *33*, 2380-2387.

[6] Chemcraft – Graphical Software for Visualization of Quantum Chemistry Computations. Https://www.chemcraftprog.com

[7] Lu, T., Chen, F., Multiwfn: a multifunctional wavefunction analyzer. *J. Comput. Chem.* **2012**, *33*, 580−592.

Crystallographic details.

Low-temperature diffraction data were collected on a Bruker D8 Quest APEX-III single crystal diffractometer with a Photon III detector and a IμS 3.0 microfocus X-ray source at the Instituto de Investigaciones Químicas, Sevilla. Data were collected by means of ω and φ scans using monochromatic radiation λ(Mo Kα1) = 0.71073 Å. The diffraction images collected were processed and scaled using APEX-4 v2021.4-0 software. The structures were solved with SHELXT and was refined against F2 on all data by full-matrix least squares with SHELXL [1], using Olex2 [2] as graphical interface. All non-hydrogen atoms were refined anisotropically. Hydrogen atoms were included in the model at geometrically calculated positions and refined using a riding model, unless otherwise noted. The isotropic displacement parameters of all hydrogen atoms were fixed to 1.2 times the U value of the atoms to which they are linked (1.5 times for methyl groups).

Crystal Data for **^{tBu}N6** $C_{28}H_{34}N_6$ (M =454.61 g/mol): triclinic, space group P-1 (no. 2), a = 6.2515(12) Å, b = 12.540(2) Å, c = 16.697(3) Å, α = 104.889(7)°, β = 93.318(7)°, γ = 104.415(7)°, V = 1214.8(4) Å3, Z = 2, T = 193.00 K, μ(MoKα) = 0.076 mm-1, Dcalc = 1.243 g/cm3, 4451 reflections measured (3.7° ≤ 2Θ ≤ 50.958°), 4451 unique (Rint = ?, Rsigma = 0.0356) which were used in all calculations. The final R1 was 0.1073 (I > 2σ(I)) and wR2 was 0.2812 (all data).

Crystal Data for **1·BF$_4$** $C_{35}H_{44.5}B_2Cu_2F_8N_{9.5}$ (*M* =837.42 g/mol): monoclinic, space group C2/c (no. 15), *a* = 19.1024(11) Å, *b* = 20.1459(11) Å, *c* = 13.8031(8) Å, *β* = 128.279(2)°, *V* = 4169.9(4) Å^3, *Z* = 4, *T* = 193.00 K, μ(MoKα) = 1.087 mm^{-1}, *Dcalc* = 1.334 g/cm^3, 61342 reflections measured (4.044° ≤ 2Θ ≤ 56.64°), 5202 unique (R_{int} = 0.1009, R_{sigma} = 0.0424) which were used in all calculations. The final R_1 was 0.0360 (I > 2σ(I)) and wR_2 was 0.1065 (all data).

Crystal Data for **2** $C_{138.5}H_{168}B_4Cl_4Cu_4F_{16}N_{24}$ (*M* =2912.16 g/mol): triclinic, space group P-1 (no. 2), *a* = 14.1082(9) Å, *b* = 21.6344(16) Å, *c* = 22.6538(16) Å, *α* = 92.791(3)°, *β* = 90.088(2)°, *γ* = 90.291(2)°, *V* = 6906.1(8) Å^3, *Z* = 2, *T* = 193.00 K, μ(MoKα) = 0.766 mm^{-1}, *Dcalc* = 1.400 g/cm^3, 427839 reflections measured (3.922° ≤ 2Θ ≤ 56.744°), 34475 unique (R_{int} = 0.0842, R_{sigma} = 0.0368) which were used in all calculations. The final R_1 was 0.0578 (I > 2σ(I)) and wR_2 was 0.1569 (all data).

Crystal Data for **3** $C_{32}H_{40}BClCu_2F_4N_8$ (*M* =786.06 g/mol): orthorhombic, space group Fdd2 (no. 43), *a* = 22.6678(10) Å, *b* = 15.2646(7) Å, *c* = 19.5928(8) Å, *V* = 6779.4(5) Å^3, *Z* = 8, *T* = 193.00 K, μ(MoKα) = 1.392 mm^{-1}, *Dcalc* = 1.540 g/cm^3, 53356 reflections measured (5.496° ≤ 2Θ ≤ 61.1°), 5178 unique (R_{int} = 0.0534, R_{sigma} = 0.0287) which were used in all calculations. The final R_1 was 0.0277 (I > 2σ(I)) and wR_2 was 0.0743 (all data).

[1] Sheldrick, G. M. Crystal structure refinement with SHELXL. *Acta Cryst.* **2008**, *A64*, 112-122.
[2] Dolomanov, O.V., Bourhis, L.J., Gildea, R.J, Howard, J.A.K. & Puschmann, H. OLEX2: a complete structure solution, refinement and analysis program. *J. Appl. Cryst.* **2009**, *42*, 339-341.
